# Toxin-Antitoxin Systems in the Mobile Genome of *Acidithiobacillus ferrooxidans*

**DOI:** 10.1371/journal.pone.0112226

**Published:** 2014-11-10

**Authors:** Paula Bustamante, Mario Tello, Omar Orellana

**Affiliations:** 1 Programa de Biología Celular y Molecular, ICBM, Facultad de Medicina, Universidad de Chile, Santiago, Chile; 2 Centro de Biotecnología Acuícola, Departamento de Biología, Facultad de Química y Biología, Universidad de Santiago de Chile, Santiago, Chile; University of Manchester, United Kingdom

## Abstract

Toxin-antitoxin (TA) systems are genetic modules composed of a pair of genes encoding a stable toxin and an unstable antitoxin that inhibits toxin activity. They are widespread among plasmids and chromosomes of bacteria and archaea. TA systems are known to be involved in the stabilization of plasmids but there is no consensus about the function of chromosomal TA systems. To shed light on the role of chromosomally encoded TA systems we analyzed the distribution and functionality of type II TA systems in the chromosome of two strains from *Acidithiobacillus ferrooxidans* (ATCC 23270 and 53993), a Gram-negative, acidophilic, environmental bacterium that participates in the bioleaching of minerals. As in other environmental microorganisms, *A. ferrooxidans* has a high content of TA systems (28-29) and in twenty of them the toxin is a putative ribonuclease. According to the genetic context, some of these systems are encoded near or within mobile genetic elements. Although most TA systems are shared by both strains, four of them, which are encoded in the active mobile element ICE*Afe*1, are exclusive to the type strain ATCC 23270. We demostrated that two TA systems from ICE*Afe*1 are functional in *E. coli* cells, since the toxins inhibit growth and the antitoxins counteract the effect of their cognate toxins. All the toxins from ICE*Afe*1, including a novel toxin, are RNases with different ion requirements. The data indicate that some of the chromosomally encoded TA systems are actually part of the *A. ferrooxidans* mobile genome and we propose that could be involved in the maintenance of these integrated mobile genetic elements.

## Introduction

Toxin-antitoxin (TA) systems are small genetic modules widely distributed in bacteria and archaea [1] that are comprised of a pair of genes encoding a stable toxin and an unstable antitoxin capable of inhibiting toxin activity [1], [2]. In contrast to bacteriocins [3] and toxins from contact-dependent inhibition systems [4], TA toxins are not secreted and inhibit cell growth by targeting key molecules in essential cellular processes such as DNA replication, mRNA stability or protein, cell-wall or ATP biosynthesis [1].

TA systems were first discovered as systems that contribute to plasmid maintenance by a phenomenon denoted as “post-segregational killing” or “addiction” [5], [6]. When a plasmid encoding a TA system is lost from a cell, the toxin is released from the existing TA complex as the unstable antitoxin decays, resulting in cell growth inhibition and eventually death [7]. In addition to plasmids, TA systems are also found in bacterial chromosomes, particularly in free-living prokaryotic cells [8], [9], but their function is not well understood [10]. Although chromosomal TA systems are not essential for normal cell growth [11], it is believed that they play key roles in stress response [12], persister phenotype [13] and stabilization of horizontally acquired genetic elements [14].

Five types of TA systems have been proposed to date. All of them comprise a toxic protein (toxin) and an antitoxin that can be either a small non-coding RNA (type I and type III [15], [16]) or a low molecular weight protein (types II, IV and V [17]–[19]). Recent studies have identified an ever-increasing number of experimentally defined, or putative, type I, type II and type III TA systems [8], [9], [15], [16]. On the other hand, type IV and type V TA systems were recently discovered and to date have only a few representatives [17], [18], [20], [21].

Type II TA systems, the most well known and the interest of this work, are encoded in operons consisting of genes that overlap (or are a few bases apart); the toxin and its cognate antitoxin form a stable protein TA complex that prevents the toxic effect [1]. Type II TA systems are diverse and are classified in 12 toxin and 20 antitoxin super-families based on sequence similarity [19]. Targets of type II toxins are also diverse, most frequently acting to cleave mRNA at specific sequences to inhibit translation in a ribosome-dependent or independent manner [22], [23].

Type II systems are thought to move from one genome to another by horizontal gene transfer (HGT) [9]. In fact, some TA systems (besides plasmidial TA) are localized within mobile genetic elements (MGEs) such as transposons and superintegrons [24], [25]. Chromosomally encoded TA systems have also been shown to have a role in the stabilization of large genomic fragments and integrative-conjugative elements (ICEs) [14], [26]. Thus, it is possible that TA systems considered to be chromosomally encoded could actually be associated with active or inactive integrated genetic elements.

The number of type II TA systems in an organism varies greatly, not only from one bacterial species to another, but also between isolates from the same species [9], [19]. Most of the organisms that have many TA systems grow in nutrient-limited environments and/or are chemolithoautotrophs (although a high TA content is observed in some obligate intracellular bacterial genomes [19]), leading to the proposal that these systems might be beneficial for this type of slow-growing microorganisms [9].

*Acidithiobacillus ferrooxidans* is an environmental acidophilic, chemolithoautotrophic Gram-negative γ-proteobacterium (although some discrepancies exist concerning its classification in this bacterial class [27]) that obtains its energy from the oxidation of ferrous ions or reduced sulfur compounds [28]. It belongs to the consortium of microorganisms that participate in the bioleaching of minerals, being a model organism for the study of bioleaching, metabolic and genomic studies of acidophilic bacteria [28], [29]. Although no genetic system has been developed for this microorganism, the genome sequences of two strains are available in public databases (ATCC 23270 and ATCC 53993 strains). A number of MGE-related DNA sequences have been described in its genome as insertion sequence elements, transposons and plasmids [28], [30], [31], including a large genomic island [32] and an actively excising integrative-conjugative element (ICE*Afe*1) [33]. As these MGEs are stably integrated into the chromosome of *A. ferrooxidans* and a number of TA-related proteins have been annotated in the genome of the two sequenced strains [28], it is possible that this environmental bacterium relies on TAs to avoid the loss of these mobile elements.

To shed light into the role of chromosomally encoded TA systems from *A. ferrooxidans* and their relation with MGEs, we studied the distribution of type II TA systems in the two available sequenced genomes in public databases. We also studied the functionality of the systems encoded in the actively excising ICE*Afe*1. Based on our data we propose that type II TA systems from *A. ferrooxidans* could be part of its mobile genome and might be involved in the maintenance of its MGEs.

## Materials and Methods

### Bioinformatic analysis

*In silico* screening for type II TA systems in *A. ferrooxidans* ATCC 23270 (NCBI RefSeq NC_011761) and ATCC 53993 (NCBI RefSeq NC_011206) was conducted using the web-based search tool TADB (http://bioinfo-mml.sjtu.edu.cn/TADB/) [34], an online resource of type II TA loci-relevant data from 'wet' experimental data as well as information garnered by bioinformatics analyses. We also used the data from RASTA-Bacteria (http://genoweb1.irisa.fr/duals/RASTA-Bacteria/) [35], an automated method allowing identification of TA loci in sequenced prokaryotic genomes, whether they are annotated open reading frames or not.

The classification of putative toxin and antitoxins in super-families was according to Leplae et al. [19]. Using BLASTP, each putative toxin and antitoxin from *A. ferrooxidans* was compared against the sequences of toxins and antitoxins from the different super-families described by Leplae et al. [19], either ‘original’, ‘similar’ or validated sequences. An E-value score threshold of 0.001 and 50% query residues aligned were used to select candidates. Each toxin or antitoxin was assigned to the super-family with the best hit and a name was given according to the best protein hit. Protein structure predictions were assayed by Phyre 2.0 server [36].

The Integrated Microbial Genomes platform (IMG, http://img.jgi.doe.gov/cgi-bin/w/main.cgi) [37] was used for the visualization of genome contexts and characteristics of each gene and protein.

### Phylogenetic analysis

Multi-alignment between nucleotide sequences encoding TA toxins was performed using ClustalW [38]. The parameters were set up to align codons using Gonnet as substitution matrix [39]. The evolutionary history was inferred using the Neighbor-Joining method [40]. The optimal tree with the sum of branch length  =  380.7 is shown. The confidence probability (multiplied by 100) that the interior branch length is greater than 0, as estimated using the bootstrap test (1000 replicates), is shown next to the branches [41]. The evolutionary distances were computed using the Maximum Composite Likelihood method [42]. The rate of variation among sites was modeled with a gamma distribution (shape parameter  =  1). The analysis involved 72 nucleotide sequences. All ambiguous positions were removed for each sequence pair. There were a total of 618 positions in the final dataset. Multialignment and evolutionary analyses were conducted in MEGA5 [43].

### Bacterial strains and growth conditions

*Escherichia coli* JM109 strain was used for cloning and plasmid maintenance. *E. coli* BL21(DE3)pLysS strain was used for recombinant protein expression and BL21(DE3) strain for plasmid maintenance tests. The strains were grown in Luria-Bertani (LB) or on LB agar at 37°C with 1% glucose. When appropriate, media were supplemented with ampicillin (100 µg/ml) or chloramphenicol (34 µg/ml). When both antibiotics were used together, they were added to half of the concentration.

### Cloning of TA systems

Toxin and antitoxin genes were amplified by PCR using PfuUltra II Fusion HS DNA Polymerase (Agilent Technologies), *A. ferrooxidans* ATCC 23270 chromosomal DNA as a template and the oligonucleotides indicated in Table 1. The pETDuet-1 expression vector (Novagen) was used for cloning. The amplified genes and vector DNA were double digested with *Bam*HI/*Hind*III or *Nde*I/*Xho*I according to the protocols indicated by the manufacturer (ThermoScientific), ligated with T4 DNA Ligase (New England Biolabs), and used to transform *E. coli* JM109 by a chemical method [44]. Three types of recombinant vectors were constructed: pETDuet-T, with toxin genes cloned into multiple cloning site-1 (MCS1) so that the toxins are expressed as N-terminal (His)_6_-tagged proteins; pETDuet-A, with the antitoxin genes cloned into MCS1; and pETDuet-TA, corresponding to pETDuet-T vectors with the cognate antitoxin genes cloned into MCS2. Transformants were selected with ampicillin and checked by colony PCR with the oligonucleotides indicated in Table 1. Cloned genes were analyzed by DNA sequencing (Macrogen, USA). Recombinant plasmids were used for transformation of *E. coli* BL21(DE3)pLysS cells by a chemical method [44].

**Table 1:** Oligonucleotides used.

Name	Sequence 5’-3’	Use
HtoxinDuet-F	AGA TCT TCT GAT GGG CGC TGC	Forward oligonucleotide for cloning the toxin gene from MazEF-1 system on pGEM-T Easy and further sub-cloning into the MCS1 from pETDuet-1.
HtoxinDuet-R	AAG CTT CTC CCA ATA GCT ATG CC	Reverse oligonucleotide for cloning the toxin gene from MazEF-1 system on pGEM-T Easy and further sub-cloning into the MCS1 from pETDuet-1.
AntitoxinDuet-F	ACC ATA TGC GGG TGA TTG TG	Forward oligonucleotide for cloning the toxin gene from MazEF-1 system on pGEM-T Easy and further sub-cloning into the MCS2 from pETDuet-1.
AntitoxinDuet-R	ATC TCG AGC GCC CAT CAG AG	Reverse oligonucleotide for cloning the toxin gene from MazEF-1 system on pGEM-T Easy and further sub-cloning into the MCS1 from pETDuet-1.
AFE1361_NdeI	GCC AGA GGC ATA TGA TTA CAA TG	Forward oligonucleotide for cloning of the antitoxin gene from StbC/VapC-3 system into the MCS2 from pETDuet-1
AFE1361_XhoI	GGT CTC GAG CAA AAT CAT GC	Reverse oligonucleotide for cloning of the antitoxin gene from StbC/VapC-3 system into the MCS2 from pETDuet-1
AFE1362_BamHI	ATA GGA TCC CAT GAT TTT GCT GG	Forward oligonucleotide for cloning of the toxin gene from StbC/VapC-3 system into the MCS1 from pETDuet-1
AFE1362_HindIII	CAT TAA GCT TGT CTC ATG TCT C	Reverse oligonucleotide for cloning of the toxin gene from StbC/VapC-3 system into the MCS1 from pETDuet-1
AFE1367_NdeI	TGT GCA TAT GCT TGA TAA GC	Oligonucleotide forward for cloning of the antitoxin gene from TA system number 9 into the MCS2 from pETDuet-1
AFE1367_XhoI	TCT CTC GAG TTG CGC ATC AAC	Reverse oligonucleotide for cloning of the antitoxin gene from TA system number 9 into the MCS2 from pETDuet-1
AFE1368_BamHI	GGG GAT CCG AAA TTT TTA GTT G	Forward oligonucleotide for cloning of the toxin gene from TA system number 9 into the MCS1 from pETDuet-1
AFE_1368_HindIII	CGA TAA GCT TCT TCA CTG ATG G	Reverse oligonucleotide for cloning of the toxin gene from TA system number 9 into the MCS1 from pETDuet-1
AFE1383_NdeI	CAT CCA TAT GAG CGG TGG CAA TG	Forward oligonucleotide for cloning of the antitoxin gene from EcoA1/EcoT1-1 system into the MCS2 from pETDuet-1
AFE1383_XhoI	CGA TCT CGA GTC ATA GCG CAC	Reverse oligonucleotide for cloning of the antitoxin gene from EcoA1/EcoT1-1 system into the MCS2 from pETDuet-1
AFE1384_BamHI	GCA GGA TCC TTT GCT CTG GGT G	Forward oligonucleotide for cloning of the toxin gene from EcoA1/EcoT1-1 system into the MCS1 from pETDuet-1
AFE1384_HindIII	GAC ATA AGC TTC GCT CAT CTC G	Reverse oligonucleotide for cloning of the toxin gene from EcoA1/EcoT1-1 system into the MCS1 from pETDuet-1
pET Upstream Primer	ATG CGT CCG GCG TAG A	Oligonucleotide for sequencing genes inserted into MCS1 from pETDuet-1
DuetDOWN-1 Primer	GAT TAT GCG GCC GTG TAC AA	Oligonucleotide for sequencing genes inserted into MCS1 from pETDuet-1
DuetUP2 Primer	TTG TAC ACG GCC GCA TAA TC	Oligonucleotide for sequencing genes inserted into MCS2 from pETDuet-1 and pACYCDuet-1
T7 Terminator Primer	GCT AGT TAT TGC TCA GCG G	Oligonucleotide for sequencing genes inserted into MCS2 from pETDuet-1 and pACYCDuet-1

For the plasmid maintenance test (see below) we constructed pACYCDuet-A plasmids, corresponding to the pACYCDuet-1 vector (Novagen) with antitoxin genes cloned into its MCS2. DNA fragments containing the antitoxin genes were obtained from the corresponding pETDuet-TA plasmids double digested with *Nde*I/*Xho*I. The fragments were ligated to pACYCDuet-1 double digested with the same enzymes and the constructs were used to transform *E. coli* JM109 by a chemical method [44]. Transformants were selected with chloramphenicol and checked by colony PCR with the oligonucleotides indicated in Table 1. Recombinant plasmids were used for transformation of *E. coli* BL21(DE3) cells by a chemical method [44].

### Evaluation of toxicity in *E. coli*

The toxicity of toxin proteins in *E. coli* BL21(DE3)pLysS was determined by the growth pattern of cultures on liquid and solid media in the presence or absence of the inducer IPTG. Overnight cultures of *E. coli* BL21(DE3)pLysS cells with plasmids containing toxin, antitoxin or both genes of each TA system were diluted 100-fold and grown in LB broth until an OD_600_ of 0.2-0.3. At this point, 1 mM IPTG was added and growth was monitored by measuring OD_600_ of the cultures in a microplate spectrophotometer (Epoch). Three hours after the induction, aliquots of each culture were 10-fold serial diluted, and 5 µl of each dilution were spotted on LB agar without IPTG and growth at 37°C for 16 hours. In addition, following the induction with IPTG, a viability assay was performed. At different time intervals culture samples were serially diluted (10-fold) and aliquots were seeded on LB plates to determine the number of colony-forming units (CFU/ml).

### Plasmid maintenance test

*E. coli* BL21(DE3) was double-transformed with the corresponding pETDuet-T and pACYCDuet-A or pETDuet-1 and pACYCDuet-A vectors. With these cultures a plasmid maintenance test was performance as in [45].

### Protein expression and purification of (His)_6_-toxins

Toxins were expressed in *E. coli* BL21(DE3)pLysS carrying the corresponding plasmids after induction with 1 mM IPTG for three hours and purified by Ni^+2^-affinity chromatography.

(His)_6_-MazF-1 was purified under native conditions from *E. coli* carrying pETMazF-1. The cells were harvested by centrifugation at 3800 g at 4°C for 10 minutes, resuspended in native lysis buffer (50 mM NaH_2_PO_4_, 300 mM NaCl, 20 mM imidazole, pH 8.0) with 1 mM PMSF and subjected to lysis by sonication. The protein extract was cleared by centrifugation at 15350 g at 4°C for 30 minutes and the supernatant was applied to a column containing 500 µl of Ni^+2^-Sepharose resin (GE Healthcare). The resin was washed with 30 column volumes of washing buffer (50 mM NaH_2_PO_4_, 300 mM NaCl, 150 mM imidazole, pH 8.0) and the retained proteins were eluted with the same buffer containing 250 mM imidazole. The purified proteins were dialyzed against storage buffer (25 mM Tris-HCl pH 8.0, 100 mM NaCl, 20% glycerol, 0.5 mM dithiothreitol) at 4°C for 16 hours followed by a second dialysis for 4 hours against fresh storage buffer and stored at −20°C.

(His)_6_-VapC-3, (His)_6_-tox28 and (His)_6_-EcoT1-1 were purified from *E. coli* carrying the corresponding pETDuet-TA plasmids. Cells were harvested by centrifugation at 3800 g at 4°C for 10 minutes, resuspended in denaturing lysis buffer (100 mM NaH_2_PO_4_, 10 mM Tris-HCl, 6 M GuHCl, pH 8.0) and incubated at ambient temperature for 1 h with agitation to achieve the TA complexes dissociation. Protein extracts were cleared by centrifugation at 15350 g at ambient temperature for 30 minutes and the supernatant applied to a column containing 500 µl of Ni^+2^-Sepharose resin (GE Healthcare). The resin was washed with 30 column volumes of denaturing wash buffer (100 mM NaH_2_PO_4_, 10 mM Tris-HCl, 8 M urea, 20 mM imidazole, pH 8.0). The elution of bound proteins was achieved by increasing the imidazole concentration in the buffer to 50 mM (for (His)_6_-VapC-3 and (His)_6_-tox28) or 100 mM (for (His)_6_-EcoT1-1). The purified proteins were refolded by dialysis against storage buffer as before and stored at -20°C.

All proteins were quantified by the method of Bradford (Bio-Rad Protein Assay) in a microplate spectrophotometer (Epoch), analyzed by Tricine-SDS-PAGE [46] and visualized by staining with Coomassie brilliant blue.

### RNase activity

The digestion reaction mixture (20 µl) consisted of 1.6 µg of MS2 RNA substrate (Roche) in 10 mM Tris-HCl (pH 7.8) with or without 10 mM MgCl_2_ or MnCl_2_, 40 U RNase inhibitor Ribolock (ThermoScientific) and 100 pmol of each purified toxin. Parallel reactions with 12 mM EDTA were used as controls. The reactions were incubated for 15 or 30 minutes at 37°C and stopped by adding 4 µl of 6X electrophoresis loading buffer. The reaction products were run on a 1% agarose gel (in 1X TAE) and visualized by staining with GelRed (Biotium).

## Results

### Content of type II TA systems in *A. ferrooxidans*

To further understand the role of chromosomally encoded TA systems in environmental microorganisms, we searched for type II TA systems (hereafter named as TA) in the publicly available genome sequences from two strains (ATCC 23270 and ATCC 53993) of the bioleaching bacterium *A. ferrooxidans*.

**Figure 1:**
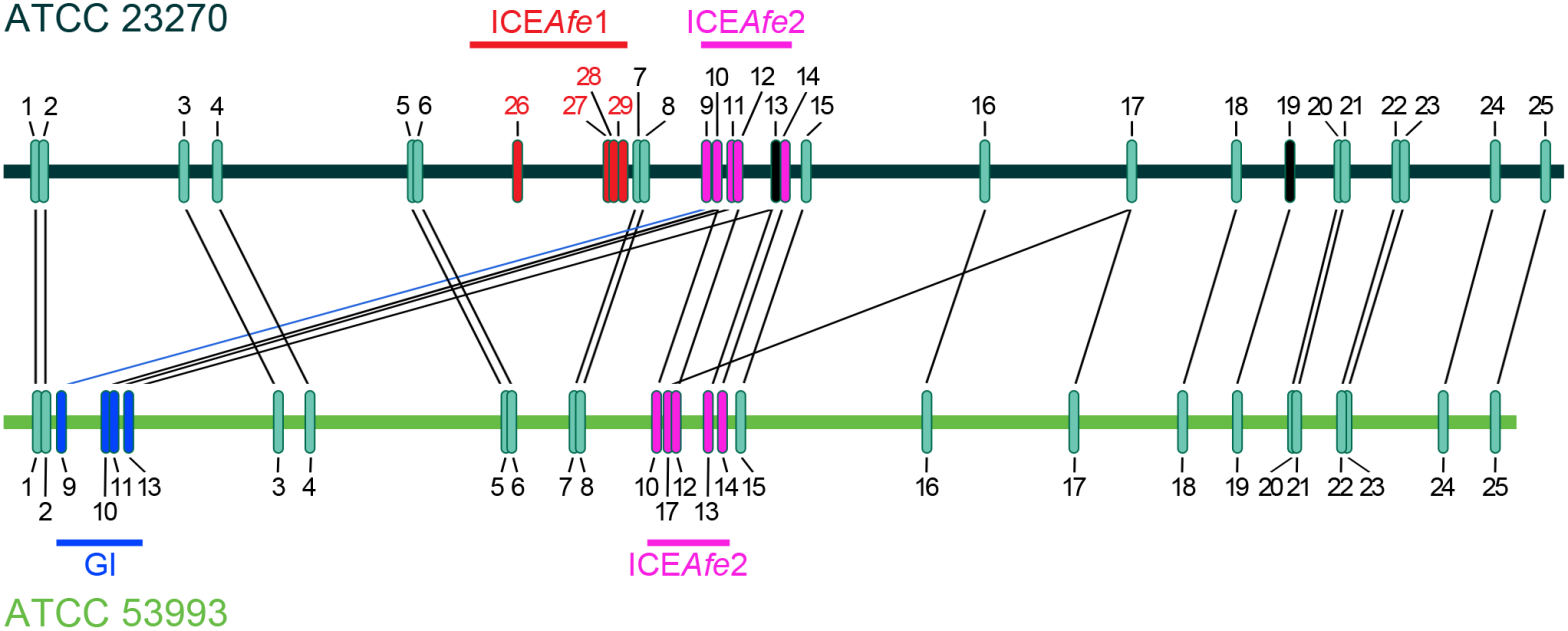
Comparison of the relative genomic locations of *A. ferrooxidans* TA systems. Using BLASTP, TA from each *A. ferrooxidans* genome were paired according to protein similarity. TA encoded in MGEs are shown in red (ICE*Afe*1), pink (ICE*Afe*2) and blue (Genomic island, GI). In black are shown TA in which the gene that must encode the toxin are pseudo genes. Black lines link TA that have 94-100% amino acid identity between the two strains. The blue line links a TA that has 49% (antitoxin) and 52% (toxin) amino acid identity with its counterpart in the other strain. Numbers of the TA are according to Table 2.

To identify shared TA between both strains, BLASTP searches were conducted, using toxin and antitoxin protein sequences from one strain as query (based on the information available in TADB) to search the proteins encoded by the other strain. From this analysis, 29 TA are encoded in *A. ferrooxidans* ATCC 23270 (including TA 13 and 19 in which the toxin gene corresponds to a pseudogene; Table 2 and Figure 1) and 28 in ATCC 53993 (including TA 10, 13 and 17 which have two identical copies; Table 2 and Figure 1). A total of 13 new putative TA were identified that were either not assigned or erroneously assigned by TADB (Supporting information S1). In support of this, we note that *A. ferrooxidans* has a high TA content and it is expected that this characteristic is shared with other bioleaching bacteria. When we analyzed the TA content of other sequenced acidophilic bioleaching bacteria with the RASTA-Bacteria platform (because they are not available in TADB), we found that *A. caldus* SM-1, *Leptospirillum ferriphilum* ML-04, *L. ferrooxidans* C2-3 and *A. ferrivorans* SS3 encode at least, 30, 16, 29 and more than 50 putative TA, respectively (data not shown).

**Table 2:** Putative type II TA systems encoded on *A. ferrooxidans* §.

TA	Antitoxin locus	Accession gi	Hits in CDD	Antitoxin super-family^a^ (name)	Toxin locus	Accession gi	Hits in CDD	Toxin super-family^a^ (name)	Chromosomal or in a MGE^b^
1	AFE_0085	218668122	Phd super family [cl18766]; COG4118	Phd (Phd-1)	AFE_0086	218666107	PIN_MT3492 [cd09874]	NI (tox1)	Chr
	Lferr_0087	198282235	Phd super family [cl18766]; COG4118	Phd (Phd-1)	Lferr_0088	198282236	PIN_MT3492 [cd09874]	NI (tox1)	Chr
2	AFE_0089	218666717	HTH_XRE [cd00093]	HigA (HigA-1)	AFE_0088	218665352	Plasmid_killer super family [cl01422]	RelE/ParE (HigB-1)	Chr
	Lferr_0091	198282239	HTH_XRE [cd00093]	HigA (HigA-1)	Lferr_0090	198282238	Plasmid_killer super family [cl01422]	RelE/ParE (HigB-1)	Chr
3	AFE_0414	218665317	Phd super family [cl18766]; COG4118	Phd (Phd-2)	AFE_0413	218666088	PIN_Sll0205 [cd09872]	VapC (VapC-1)	Chr
	Lferr_0577	198282717	Phd super family [cl18766]; COG4118	Phd (Phd-2)	Lferr_0576	198282716	PIN_Sll0205 [cd09872]	VapC (VapC-1)	Chr
4	AFE_0477	218665855	VagC super family [cl18787]	VapB (VapB-1)	AFE_0478	218667984	PIN_VapC-FitB [cd09881]	VapC (VapC-2)	Chr
	Lferr_0637	198282777	VagC super family [cl18787]	VapB (VapB-1)	Lferr_0638	198282778	PIN_VapC-FitB [cd09881]	VapC (VapC-2)	Chr
5	AFE_0869	218667933	PhdYeFM_antitox [pfam02604]	NI (antitox5)	AFE_0870	218667345	Plasmid_Txe super family [cl17389]	RelE/ParE (YoeB-1)	Chr
	Lferr_0994	198283126	PhdYeFM_antitox [pfam02604]	NI (antitox5)	Lferr_0995	198283127	Plasmid_Txe super family [cl17389]	RelE/ParE (YoeB-1)	Chr
6	AFE_0889	218667173	PhdYeFM_antitox super family [cl09153]	Phd (StbD-1)	AFE_0890	218665915	RelE [COG2026]	RelE/ParE (StbE-1)	Chr
	Lferr_1011	198283139	PhdYeFM_antitox super family [cl09153]	Phd (StbD-1)	Lferr_1012	198283140	RelE [COG2026]	RelE/ParE (StbE-1)	Chr
7	AFE_1413	218667280	PRK09974; AbrB [COG2002]	NI (antitox7)	AFE_1412	218665529	Toxin_YhaV [pfam11663]	NI (tox7)	Chr
	Lferr_1133	198283260	PRK09974; AbrB [COG2002]	NI (antitox7)	Lferr_1132	198283259	Toxin_YhaV [pfam11663]	NI (tox7)	Chr
8	AFE_1418	218665082	COG4453	NI (antitox8)	AFE_1417	218665278	Acetyltransf_1 [pfam00583]	NI (tox8)	Chr
	Lferr_1137	198283264	COG4453	NI (antitox8)	Lferr_1136	198283263	Acetyltransf_1 [pfam00583]	NI (tox8)	Chr
9	AFE_1560	218667662	DUF4415 [pfam14384]	NI (antitox9)	AFE_1559	218665905	NI	NI (tox9)	ICE*Afe*2-23270
	Lferr_0133	198282280	DUF4415 [pfam14384]	NI (antitox9)	Lferr_0132	198282279	NI	NI (tox9)	GI
10	AFE_1579	218667623	DUF2191 [pfam09957]	RelB (RelB-1)	AFE_1578	218666342	VapC [COG1487]	NI (tox10)	ICE*Afe*2-23270
	Lferr_0230	198282374	DUF2191 [pfam09957]	RelB (RelB-1)	Lferr_0229	198282373	VapC [COG1487]	NI (tox10)	ICE*Afe*2-53993
	Lferr_1290	198283414	DUF2191 [pfam09957]	RelB (RelB-1)	Lferr_1289	198283413	VapC [COG1487]	NI (tox10)	GI
11	AFE_1614	218666352	Excise [TIGR01764]	NI (antitox11)	AFE_1613	218668111	PIN_3 [pfam13470]	NI (tox11)	ICE*Afe*2-23270
	Lferr_0234	198282378	Excise [TIGR01764]	NI (antitox11)	Lferr_0233	198282377	PIN_3 [pfam13470]	NI (tox11)	GI
12	AFE_1631	218665941	HTH_XRE [cd00093]	NI (antitox12)	AFE_1630’		upstrm_HI1419 [TIGR02683]	NI (tox12)	ICE*Afe*2-23270
	Lferr_1332	198283452	HTH_XRE [cd00093]	NI (antitox12)	Lferr_1331	198283451	upstrm_HI1419 [TIGR02683]	NI (tox12)	ICE*Afe*2-53993
13	AFE_1700	218667519	NI	RelB (Paa1-1)	Pseudo		ParE [COG3668]	NI (tox13)	ICE*Afe*2-23270
	Lferr_0263	198282407	NI	RelB (Paa1-1)	Lferr_0264	198282408	ParE [COG3668]	NI (tox13)	ICE*Afe*2-53993
	Lferr_1399	198283518	NI	RelB (Paa1-1)	Lferr_1400	198283519	ParE [COG3668]	NI (tox13)	GI
14	AFE_1732	218667149	YcfA super family [cl00752]	NI (antitox14)	AFE_1733	218666062	UPF0150 [pfam03681]	NI (tox14)	ICE*Afe*2-23270
	Lferr_1422	198283540	YcfA super family [cl00752]	NI (antitox14)	Lferr_1423	198283541	UPF0150 [pfam03681]	NI (tox14)	ICE*Afe*2-53993
15	AFE_1779	218665129	StbC super family [cl01921]	NI (antitox15)	AFE_1780	218665399	PIN_VapC-FitB [cd09881]	VapC (VapC-4)	Chr
	Lferr_1455	198283572	StbC super family [cl01921]	NI (antitox15)	Lferr_1456	198283573	PIN_VapC-FitB [cd09881]	VapC (VapC-4)	Chr
16	AFE_2130	218665450	VagC [COG4456]	VapB (MvpA-1)	AFE_2129	218668039	PIN_VapC-FitB [cd09881]	VapC (VapC-5)	Chr
	Lferr_1789	198283896	VagC [COG4456]	VapB (MvpA-1)	Lferr_1788	198283895	PIN_VapC-FitB [cd09881]	VapC (VapC-5)	Chr
17	AFE_2415	218666500	DUF4415 [pfam14384]	NI (antitox17)	AFE_2414	218666978	DUF497 super family [cl01108]	NI (tox17)	Chr
	Lferr_1314	198283435	DUF4415 [pfam14384]	NI (antitox17)	Lferr_1315	198283436	DUF497 super family [cl01108]	NI (tox17)	ICE*Afe*2-53993
	Lferr_2046	198284147	DUF4415 [pfam14384]	NI (antitox17)	Lferr_2045	198284146	DUF497 super family [cl01108]	NI (tox17)	Chr
18	AFE_2658	218666707	DUF4415 [pfam14384]	NI (antitox18)	AFE_2657’		NI	NI (tox18)	Chr
	Lferr_2284	198284371	DUF4415 [pfam14384]	NI (antitox18)	Lferr_2283	198284370	NI	NI (tox18)	Chr
19	AFE_2771	218665366	VagC super family [cl18787]	VapB (VapB-2)	Pseudo		PIN_VapC-FitB [cd09881]	NI (tox19)	Chr
	Lferr_2392	198284478	VagC super family [cl18787]	VapB (VapB-2)	Lferr_2391	198284477	PIN_VapC-FitB [cd09881]	NI (tox19)	Chr
20	AFE_2886	218668170	Antitoxin-MazE super family [cl00877]	VapB (MazE-2)	AFE_2885	218665972	PemK [pfam02452]	CcdB/MazF (PemK-1)	Chr
	Lferr_2506	198284586	Antitoxin-MazE super family [cl00877]	VapB (MazE-2)	Lferr_2505	198284585	PemK [pfam02452]	CcdB/MazF (PemK-1)	Chr
21	AFE_2889	218666996	Antitoxin-MazE super family [cl00877]	VapB (VapB-3)	AFE_2888	218666880	PIN_VapC-Smg6-like [cd09855]	VapC (NspT2-1)	Chr
	Lferr_2509	198284589	Antitoxin-MazE super family [cl00877]	VapB (VapB-3)	Lferr_2508	198284588	PIN_VapC-Smg6-like [cd09855]	VapC (NspT2-1)	Chr
22	AFE_2981	218665144	NI	NI (antitox22)	AFE_2982	218667184	NI	RelE/ParE (CcrT1-1)	Chr
	Lferr_2595’		NI	NI (antitox22)	Lferr_2595’’		NI	RelE/ParE (CcrT1-1)	Chr
23	AFE_2983	218666488	Phd [COG4118]	NI (antitox23)	AFE_2984	218665493	PIN_MT3492 [cd09874]	NI (tox23)	Chr
	Lferr_2596	198284676	Phd [COG4118]	NI (antitox23)	Lferr_2597	198284677	PIN_MT3492 [cd09874]	NI (tox23)	Chr
24	AFE_3174	218665847	Phd super family [cl18766]	NI (antitox24)	AFE_3173	218665111	PIN_2 [pfam10130]	NI (tox24)	Chr
	Lferr_2770	198284847	Phd super family [cl18766]	NI (antitox24)	Lferr_2769	198284846	PIN_2 [pfam10130]	NI (tox24)	Chr
25	AFE_3268	218667137	PhdYeFM_antitox [pfam02604]	Phd (YefM-1)	AFE_3269	218665388	PIN_3 super family [cl17397]	NI (tox25)	Chr
	Lferr_2866	198284937	PhdYeFM_antitox [pfam02604]	Phd (YefM-1)	Lferr_2867	198284938	PIN_3 super family [cl17397]	NI (tox25)	Chr
26	AFE_1098	218667753	MazE [COG2336]	VapB (MazE-1)	AFE_1099	218666923	PemK super family [cl00995]	CcdB/MazF (MazF-1)	ICE*Afe*1
27	AFE_1361	218667301	StbC super family [cl01921]	NI (antitox27)	AFE_1362	218667390	PIN_VapC-FitB [cd09881]	VapC (VapC-3)	ICE*Afe*1
28	AFE_1367	218667849	DUF433 [pfam04255]	NI (antitox28)	AFE_1368	218665288	COG4634 super family [cl18792]	NI (tox28)	ICE*Afe*1
29	AFE_1383	218666557	HTH_XRE [cd00093]; HipB [COG1396]	HigA (EcoA1-1)	AFE_1384	218667318	Gp49 super family [cl01470]	RelE/ParE (EcoT1-1)	ICE*Afe*1

§ Locus, accession gi and hits in CDD are according to the NCBI.

a according to the classification by Leplae et al., [19]; NI: not identified.

b Chr: chromosomal TA II; when a TA II is encoded in a MGE, the name of the element is indicated; in the case of ICE*Afe*2, the name is followed by the number of *A. ferrooxidans* ATCC strain where the TA is present.

As is described in some TA (mainly in *higBA* family) [47]–[49], an organization opposite to the classical gene arrangement (toxin gene encoded after the antitoxin gene) was found in six systems from *A. ferrooxidans* (TA 2, 9, 12, 17, 18 and 29).

All TA from *A. ferrooxidans* ATCC 53993 have counterparts in the other strain (sharing 94-100% amino acid identity; Figure 1, TA 1-25). Strikingly, type strain ATCC 23270 contains four exclusive TA (TA 26-29), encoded in a MGE as discussed below (Figure 1, highlighted in red). As TA 1 to 25 are the same in both strains, we will refer only to TA from ATCC 23270 strain hereafter (if not otherwise indicated).

**Figure 2:**
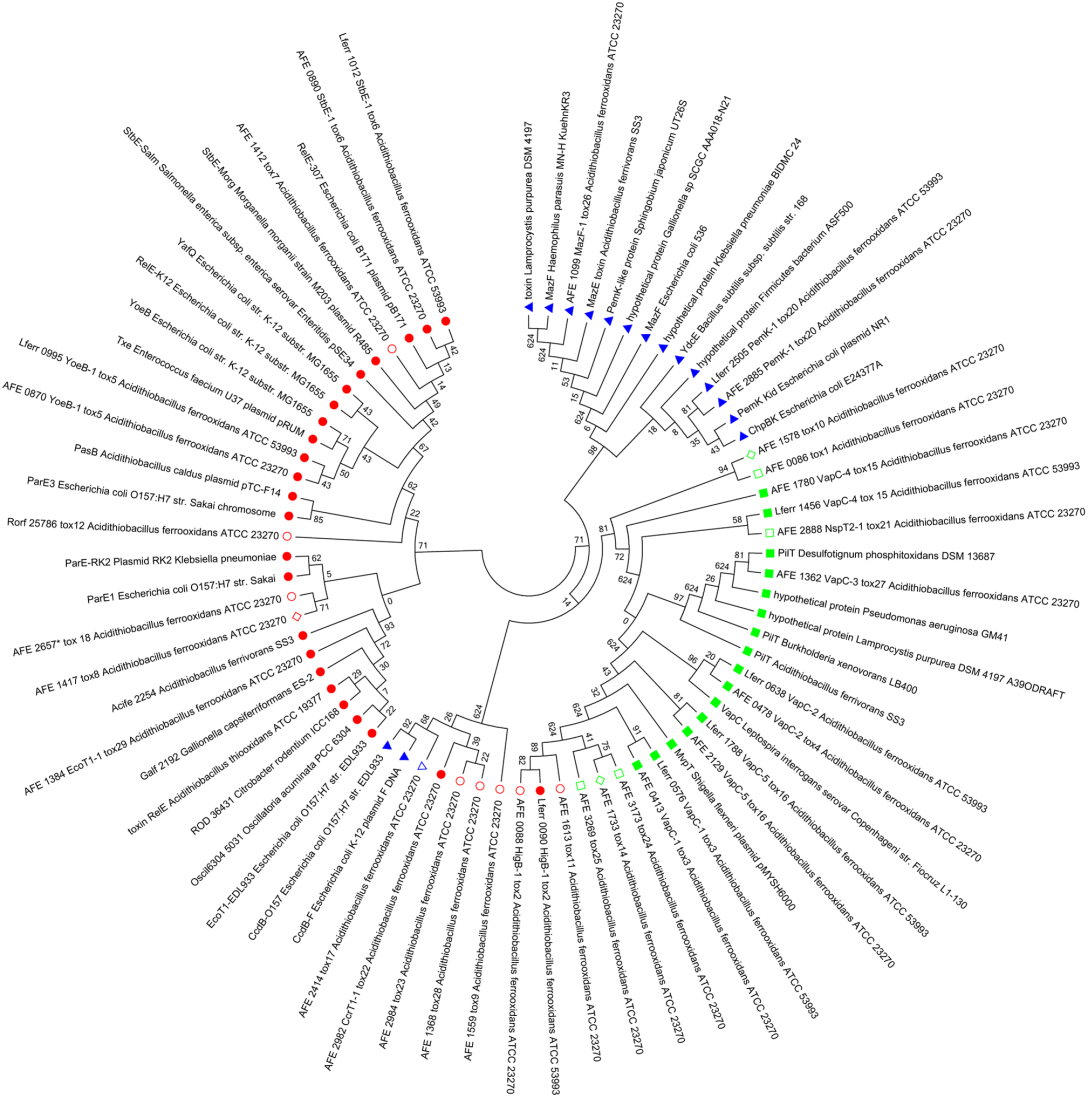
Phylogenetic relationship between TA toxins of *A. ferrooxidans* ATCC 23270. Circular unrooted dendogram built using Neighbor-Joining method. Scale shows the evolutionary distance in number of base substitutions per site. Toxins described by Leplae et al [19] belonging to RelE/ParE (red full-filled circle), CcdB/MazF (blue full-filled triangles) and VapC (green full-filled squared) super-families were introduced in the analysis as reference. Toxin classifications performed according the homologs with lower evolutionary distance (Table S1) are show in open symbols. The sequences whose homologs with lower evolutionary distance correspond to a non previously classified toxin are show in open rhomboid. The accession numbers of the sequences used in the analysis are in Supporting information S3.

Nowadays, TA systems are classified as independent toxin and antitoxin super-families instead of TA families as before [19]. Based on this classification, we assigned 14 antitoxins and 13 toxins to a given super-family according to amino acid sequence similarity (Table 2). The prevalent antitoxin super-families in *A. ferrooxidans* are Phd and VapB (4 and 5 representative of each respectively), whereas the toxins that we could assign to a super-family belong to VapC, RelE/ParE and CcdB/MazF super-families, with 6, 5 and 2 representatives each, respectively. Specifically, toxins containing PIN domains are the most abundant in *A. ferrooxidans* (thirteen TA, 48% of the toxins). It is known that TA toxins show limited sequence similarity, despite having common folds [50] and this might explain why sixteen toxins from *A. ferrooxidans* could not be assigned in the current classification. Indeed, there are seven PIN domain toxins in *A. ferrooxidans* (TA 1, 10-11, 19 and 23-25, Table 2) that do not show a suitable sequence similarity with VapC super-family proteins (those containing PIN domains). Nonetheless, these toxins show high structural homology with characterized VapC toxins (Supporting information S2) and are clustered within the VapC super-family in a phylogenetic analysis (Figure 2, green squared). Using the same phylogenetic approach, the rest of the unclassified toxins grouped within different super-families, with some of them forming a different clade (e. g. TA 1, 9, 10, 23 and 28; Figure 2, shown in open symbols).

Based on the conserved domain database (CDD [51]) hits and the super-families of each toxin identified in *A. ferrooxidans*, we predicted that twenty toxins might be ribonucleases that possibly function as translation inhibitors. Functional analysis of some of these ribonucleases associated with MGEs is described below.

### TA encoded in MGEs

TA may be associated with MGEs allowing their movement between microorganisms by HGT [25], [52]. To elucidate whether chromosomal TA systems from *A. ferrooxidans* form part of MGEs and to predict whether they have been acquired or they have the potential to be mobilized by HGT, we analyzed their genetic context.

Recently, we identified and characterized ICE*Afe*1, an active 291-kbp ICE from *A. ferrooxidans* type strain ATCC 23270; this element is excised from the chromosome of the bacterium and has the potential to be transferred by conjugation [33]. *A. ferrooxidans* ATCC 53993 also encodes a 164-kbp genomic island (GI) that provides additional copper resistance to the bacterium [32]. Further other putative ICE, ICE*Afe*2, is shared by both strains, although these elements are not identical (176-kbp and 159-kbp in ATCC 23270 and ATCC 53993, respectively). A detailed analysis of TA encoded in these MGEs revealed that ICE*Afe*1, ICE*Afe*2 and the GI contain four, five (six in ATCC 23270 including a pseudo gene) and four of them, respectively (Figure 1 highlighted in red, pink and blue). Remarkably, TA from ICE*Afe*1 (TA 26-29, Figure 1 highlighted in red) are exclusive to ATCC 23270, consistent with the unique presence of this ICE in this strain. On the other hand, ICE*Afe*2 is present in both strains and thus TA encoded within this MGE are shared (TA 9-14 and 17, Figure 1 highlighted in pink). Some of these TA are also encoded in the GI from ATCC 53993 (TA 9-11 and 13, Figure 1, highlighted in blue) suggesting a duplication of these TA systems in this strain. Interestingly, TA encoded in the GI are close to transposon-related sequences and integrases genes and have a different genomic context to their counterparts in the ICE*Afe*2 (not shown). These findings reinforce the notion that certain chromosomal TA in *A. ferrooxidans* have the potential to be mobilized by HGT and to form part of its mobile genome. Other TA in *A. ferrooxidans* are also encoded near to transposases or transposon related-genes (e.g. TA systems from ICE*Afe*1, Figure S1).

### TA from ICE*Afe*1 are functional and their toxins are ribonucleases

Because TA systems have been proposed to participate in the maintenance of MGEs, we hypothesize that TAs encoded in ICE*Afe*1, ICE*Afe*2 and the GI might contribute to prevent the loss of these elements from the *A. ferrooxidans* chromosome. Indeed, although we do not know yet the function of ICE*Afe*1 or the advantage for strain ATCC 23270 to carry it, it is stably maintained in laboratory conditions despite being unique to this strain among the other 12 strains that we have analyzed [33]. We therefore carried out a functional analysis of TA from ICE*Afe*1.

Three out of four TA from ICE*Afe*1 (TA 26, 27 and 29) share sequence similarity to well-known super-families (Table 2) and are grouped within their corresponding super-families on phylogenetic trees (Figure 2).

TA 26 (MazEF-1) is similar to the MazEF system from *E. coli* [53]–[56]. The putative toxin (MazF-1) is 51.8% identical (65.8% similar) with its counterpart from *E. coli* (Figure S2A). On the other hand, the putative antitoxin (MazE-1) is 42.7% identical (65.9% similar) to the orthologue from *E. coli* (Figure S2B). A number of conjugation genes and genes from a transposon are encoded both upstream and downstream to this TA system, respectively (Figure S1A).

TA 27 has conserved domains similar to StbC antitoxins and VapC toxins (Table 2). The toxin, VapC-3, has low sequence identity with VapC proteins but it conserves the three acidic residues from PIN-domains that are important for toxin activity (Figure S3). It is encoded near a cluster of genes that are involved in the biosynthesis and export of exopolysaccharides (Figure S1B).

TA 29 is encoded by AFE_1383/AFE_1384 genes. This system is encoded near to two other TA and close to transposition-related sequences (Figure S1B). The antitoxin is encoded by AFE_1383 and has a HTH_XRE conserved domain present on HigA and VapB antitoxins [2], [35]. Similar to the classical TA loci *higBA* [43], this TA is unusual because the toxin-encoding gene is located upstream of the antitoxin-encoding gene. The toxin encoded by AFE_1384 has a Gp49 super family conserved domain and amino acid similarity with RelE/ParE super-family. The highest amino acid identity found is with a new toxin EcoT1_EDL933_ identified and validated by Leplae et al [19], thus we named it EcoT1-1.

Phylogenetic data revealed that TA toxins 26, 27 and 29 clustered within their corresponding toxin super-families, but in a different clade from their chromosomal counterparts (Figure 2). These data reinforces the fact that these systems are part of the mobile genome from *A. ferrooxidans*.

TA 28, encoded by AFE_1367/AFE_1368 genes, has no orthologue described to date and it was ascribed as a TA system based on the characteristic of the operon by RASTA-Bacteria (Supporting information S1). On the phylogenetic analysis this toxin is closer to a putative RelE/ParE toxin from TA 22 but within a heterogeneous clade involving CcdB and not classified toxins (Figure 2). According to the information from the Integrated Microbial Genomes platform, tox28 contains a Mut7-C domain (pfam01927), which corresponds to a C-terminal RNase domain with a PIN fold [57]. Indeed, structural homology searches predicted in tox28 the presence of a putative fold like a 3-phosphoglycerate dehydrogenase (PDB ID: 2EKL; 77% coverage) and low structural homology with PIN domain proteins (PDB ID: 1O4W, 39% coverage; PDB ID: 3H87, 26% coverage). According to BLASTP results, this toxin is conserved in different species, especially in cyanobacteria and Gram-positive bacteria. Therefore, this TA system might be a novel system with an RNase toxin related to PIN domain proteins. In *A. ferrooxidans* TA 28 is encoded very close to TA 27 and transposon-related sequences (Figure S1B).

**Figure 3:**
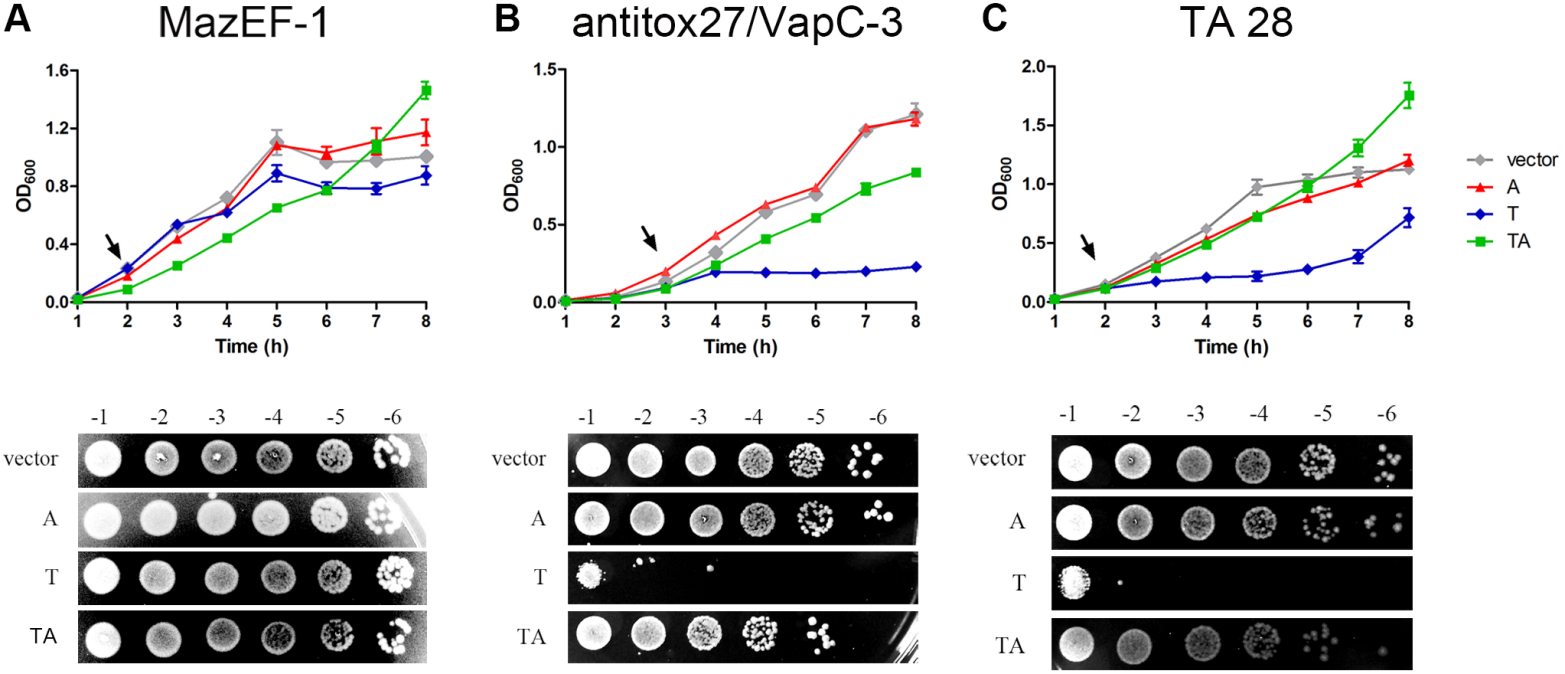
Effect of ICE*Afe*1 TA systems expression in *E. coli* growth. Cellular growth of *E. coli* BL21(DE3)pLysS cells harboring plasmids containing toxin (T, blue curves), antitoxin (A, red curves) or both (TA, green curves) genes of TA 26 (A), TA 27 (B) and TA 28 (C) was monitored by measuring the OD_600_. Cells containing the empty vector (gray curves) were used as a control. The arrows indicate the moment when 1 mM IPTG was added to each culture. 3 hours after the induction 10-fold serial dilutions of each culture were spotted on LB plates without IPTG (panels below each graph). The means and standard deviation of three different experiments are plotted.

The functionality of TA from ICE*Afe*1 was tested by transformation of *E. coli* BL21(DE3)pLysS with a multicopy plasmid (pETDuet-1 derivatives) carrying genes encoding either the toxin, the antitoxin or both (see Table 3 and Methods for a description of each plasmid). Induction of VapC-3 and tox28 expression caused cell growth arrest of *E. coli* (Figure 3, blue lines). As expected, the co-induction of cognate antitoxins restored cell growth (Figure 3, green lines). Growth of cells expressing only the antitoxins was not affected (Figure 3, red lines). Overexpression of MazF-1 did not caused cell growth arrest of *E. coli* (Figure 3A). Conversely, overexpression of VapC-3 and tox28 seems to be bactericidal. In these cases the growth is not restored when the cells are transferred to a non-inducer medium (Figure 3B and C, lower panels). These results are consistent with a decrease in the CFU count (Figure S4). On the other hand, EcoT1-1 could not be cloned in the absence of its cognate antitoxin gene, which sheds light on its high toxicity in *E. coli* cells. These results indicated that, as all these toxins are RNases (bellow), they could target different cellular RNAs and/or have different sequence specificities. VapC-3, tox28 and EcoT1-1 probably target important RNAs in *E. coli* cells, while no target (or underrepresented targets) is present for MazF-1 in this host.

**Table 3:** Plasmids used.

Plasmid	Characteristics	Reference
pGEM-T Easy	*E. coli* cloning vector. Ampicillin resistance.	Promega
pETDuet-1	*E. coli* expression vector. Ampicillin resistance. This vector is designed for the co-expression of two target genes. The vector contains two multiple cloning site (MCS1 and MCS2), each of which is preceded by a T7 promoter/lac operator and a ribosome binding site. ColE1 replicon.	Novagen
pACYCDuet-1	*E. coli* expression vector. Chloramphenicol resistance. This vector is designed for the co-expression of two target genes. The vector contains two multiple cloning site (MCS1 and MCS2), each of which is preceded by a T7 promoter/lac operator and a ribosome binding site. P15A replicon.	Novagen
pETMazE-1	pETDuet-1 derivative. Expressing the antitoxin gene from TA 26 system.	This work
pETMazF-1	pETDuet-1 derivative. Expressing the toxin gene from TA 26 system with a His_6_ tag at the N-terminal.	This work
pETMazEF-1	pETDuet-1 derivative. Expressing TA 26 system. In this construction the toxin gene has a His_6_ tag at the N-terminal.	This work
pETantitox27	pETDuet-1 derivative. Expressing the antitoxin gene from the TA 27 system.	This work
pETVapC-3	pETDuet-1 derivative. Expressing the toxin gene from TA 27 system with a His_6_ tag at the N-terminal.	This work
pETStbC-VapC-3	pETDuet-1 derivative. Expressing the TA 27 system. In this construction the toxin gene has a His_6_ tag at the N-terminal.	This work
pETantitox28	pETDuet-1 derivative. Expressing the antitoxin gene from TA 28 system.	This work
pETtox28	pETDuet-1 derivative. Expressing the toxin gene from TA 28 system with a His_6_ tag at the N-terminal.	This work
pETTA28	pETDuet-1 derivative. Expressing the TA 28 system. In this construction the toxin gene has a His_6_ tag at the N-terminal.	This work
pEcoA1/EcoT1-1	pETDuet-1 derivative. Expressing the TA 29 system. In this construction the toxin gene has a His_6_ tag at the N-terminal.	This work
pACYCantitox27	pACYCDuet-1 derivative with the antitoxin27 gene cloned at its MCS2.	This work
pACYCantitox28	pACYCDuet-1 derivative with the antitoxin28 gene cloned at its MCS2.	This work

**Figure 4:**
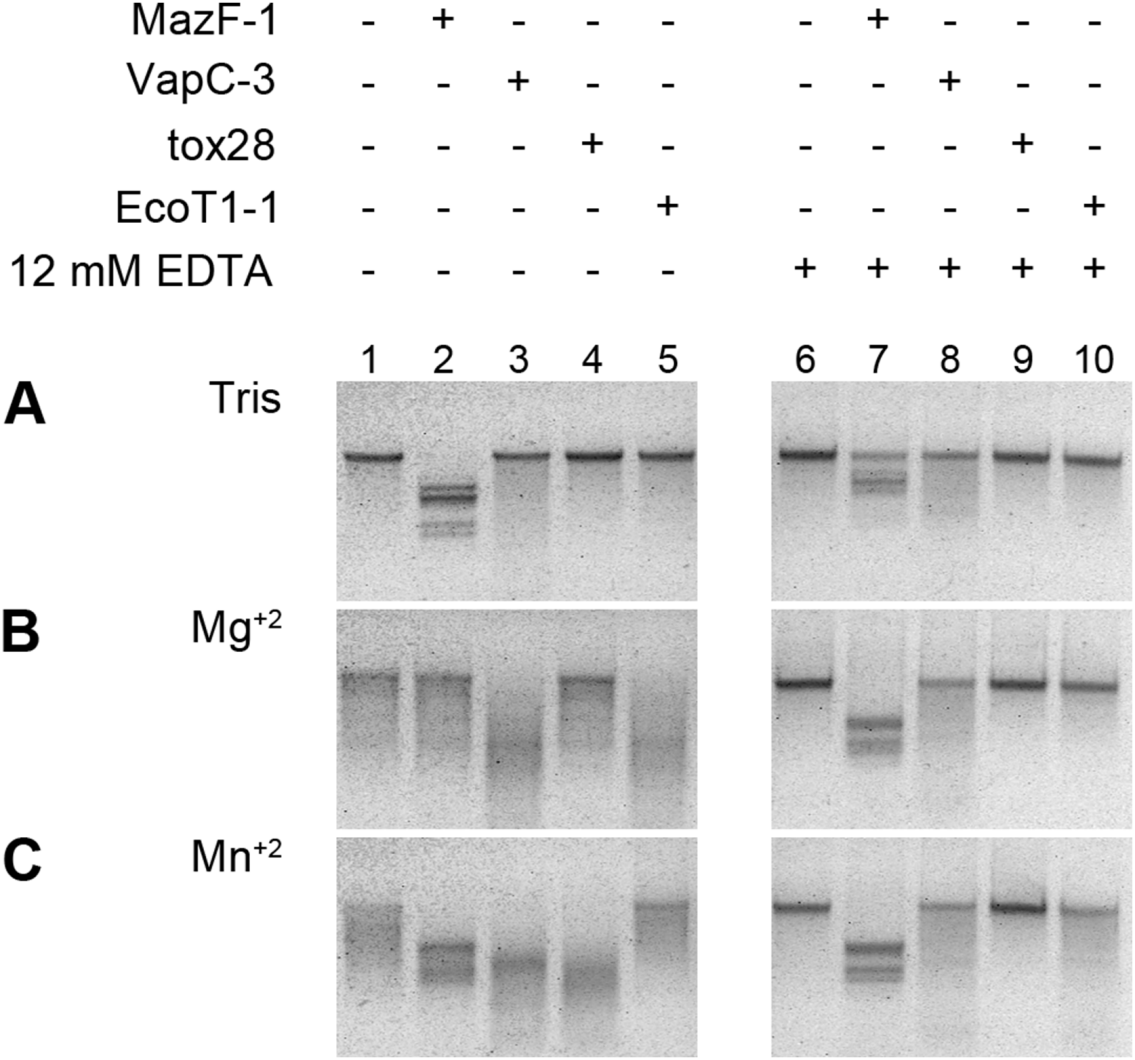
*In vitro* RNase assay of ICE*Afe*1 toxins. 1.6 µg of MS2 RNA was incubated with (+) or without (−) the purified toxins in 10 mM Tris-HCl (pH 7.8) in the absence of divalent ions (A) or with 10 mM MgCl_2_ (B) or MnCl_2_ (C). The reactions were incubated at 37°C for 15 (A and C) or 30 minutes (B). 12 mM EDTA was added to some reactions as a control (lanes 6-10).

According to bioinformatic data all these toxins were predicted to be ribonucleases. To test the ability of these proteins to hydrolyze RNA, we performed *in vitro* cleavage of viral MS2 RNA assays with purified toxins (Figure S5). MazF-1 digested the RNA only in the absence of Mg^+2^ ions (Figure 4, lane 2). Surprisingly, RNA cleavage by MazF-1 apparently is blocked by Mg^+2^ and it appears to be insensitive to Mn^+2^ ions (Figure 4B, lane 2-4), a phenomenon not yet described for a MazF toxin to our knowledge. Consistent with this, EDTA addition restored the RNase activity (Figure 4B, lane 7). Conversely, VapC-3 exhibited RNase activity only when Mg^+2^ or Mn^+2^ ions were present (Figure 4B and C, lane 3). Strikingly, under our tested conditions tox28 was active only in the presence of Mn^+2^ ions (Figure 4C, lane 4). According to our knowledge there are no TA toxins that required Mn^+2^ ions for their RNase activity. Some VapC toxins bind Mg^+2^ and/or Mn^+2^ [58] or have been crystallized with bound Mn^+2^, as in the case of VapC toxin PAE0151 from *Pyrobaculum aerophilum* [59], but its activity has been assayed only with Mg^+2^. In contrast to tox28, in the analyzed conditions EcoT1-1 was an active RNase only in the presence of Mg^+2^ ions (Figure 4B, lane 5). All Mg^+2^/Mn^+2^-dependent RNase activities were inhibited in the presence of EDTA (Figure 4, lane 6-10), confirming that the divalent ions are necessary for the activity.

Thus, three out of four TA encoded in ICE*Afe*1 (with the exception of MazF-1) are active in a heterologous system, i.e. the expression of toxins in *E. coli* arrest growth and the cognate antitoxins suppress this toxicity. Furthermore, these results show that all ICE*Afe*1 TA toxins are RNases with different ions requirements that could inhibit translation via RNA cleavage. Cellular targets and cleavage sites of these toxins are our future interest to study.

### TA systems from ICE*Afe*1 might allow plasmid maintenance

Given that TA from ICE*Afe*1 are functional and in general TA systems have been proposed to participate avoiding the loss of MGEs from their hosts, we hypothesize that these TA might be responsible of the stable maintenance of ICE*Afe*1 in *A. ferrooxidans* chromosome. To determine whether TA systems from ICE*Afe*1 mediate plasmid stability, we assay the capacity of TA 27 and TA 28 to avoid the loss of pACYCDuet-1, a vector carrying chloramphenicol resistance. We could not assay TA 26 and TA 29 given that MazF-1 (TA 26) did not affect the growth of *E. coli* (Figure 3A) and, on the other hand, the gene encoding EcoT1-1 (TA 29) could not be cloned in *E. coli*.

The assay consisted on monitoring the stability of pACYCDuet-A vectors during cultivation in the presence of selective antibiotic pressure towards pETDuet-T vectors [45]. After 30 days of culture in the absence of chloramphenicol, although they were not fully maintained (and that we obtained high standard deviations in the experiment), pACYCantitox27 and pACYCantitox28 were lost to a lower level in the absence of their cognate pETDuet-T vectors in comparison to cultures with the pETDuet-1 empty vector (Table 4). As *E. coli* BL21(DE3) contains its native chromosomal TA systems, the failure to pACYCantitox27 and pACYCantitox28 to be fully maintained, may be due to cross-interaction between ICE*Afe*1 toxins with cognate antitoxins from *E. coli*. This may have made the presence of ICE*Afe*1 antitoxins dispensable and thus the pACYCDuet-A vectors lost. Functional interaction between chromosomal and plasmidial TA systems has been demonstrated before [60]. Nevertheless, these data demonstrate that TA 27 and TA 28 encoded on a MGE from *A. ferrooxidans* could have a plasmid-stabilizing role.

**Table 4:** Plasmid maintenance test. *E. coli* BL21(DE3) was double transformed with the plasmids indicated.

	Plasmid maintenance (%)^a^			
Culture	15 days	20 days	26 days	30 days
pETVapC-3+pACYCantitox27	70.87±37.93	88.10±16.83	89.03±13.51	76.67±33.00
pETDuet-1+pACYCantitox27	52.77±33.56	44.67±27.15	44.97±22.13	35.03±31.94
pETtox28+pACYCantitox28	62.23±34.59	55.42±37.93	55.83±41.05	51.43±40.88
pETDuet-1+pACYCantitox28	64.70±44.87	60.83±43.03	40.73±29.02	28.20±21.25

a percentage of chloramphenicol-resistant bacteria (resistance gene encoded on pACYCDuet-1) when cultured on ampicillin-containing media (resistance gene encoded on pETDuet-1) for the days indicated. The data are expressed as the means of three independent cultures ± standard deviation.

## Discussion

In this study, the content of type II TA systems and their relationship with MGEs in the environmental bacterium *A. ferrooxidans* were investigated. According to the data presented here, *A. ferrooxidans* encodes at least 29 and 28 TA in ATCC 23270 and ATCC 53993 strains, respectively (representing 1.8 and 2% of total number of CDSs, respectively). Given this content of TA and considering the number of putative TA proteins that we could not classify within a super-family, it is expected that this microorganism could be a source of novel systems, expanding the repertoire currently known. It seems to be the case for TA 28 characterized in this work; a TA present in a MGE encoding a novel toxin that causes a bactericidal state in *E. coli* and has a Mn^+2^-dependent RNase activity. The described number of TA in *A. ferrooxidans* could be underestimated because of the several hypothetical proteins encoded in a TA-like gene organization as well as putative orphan toxins. Additionally, we must consider that type I, III, IV and V TA systems, that were not the subject of this study, might contribute to the total number of TA systems in *A. ferrooxidans*.

### Putative roles of ICE*Afe*1 TA systems

In the ICE SXT from *V. cholera*, the MosAT system promotes the maintenance of the element. The mRNA levels of MosAT system are enhanced when the element is excised, preventing its loss [14]. Since the levels of TA mRNAs from ICE*Afe*1 do not increase upon their excision (based on qRT-PCR, data not shown), it seems that these systems do not behave in the same way as the MosAT system. We cannot rule out that there might be changes at a protein level in different growth conditions.

Plasmid-encoded TA systems prevent the proliferation of plasmid-free progeny and thereby contribute to the maintenance of their replicons. By a similar mechanism, chromosomal genes closely linked to a TA locus could have a selective advantage; as a consequence the maintenance of specific genes (like TA genes) might have an effect on the stability and spread of MGEs. Here we have shown that two TA from *A. ferrooxidans* ICE*Afe*1 (TA 27 and TA 28) seem to have a role in the maintenance of MGEs (Figure 4). Thus, the presence of TA on MGEs in *A. ferrooxidans* could explain why they are stably maintained in this bacterium.

Based on the genomic contexts, we hypothesize that TA from ICE*Afe*1 might be involved in the conjugal transfer and/or biofilm formation, putative roles ascribed to this MGE [33]. The MazEF-1 system (TA 26) is encoded very close to the conjugation cluster probably responsible for the horizontal transfer of this element (Figure S1A). On the other hand, TA 27, TA 28 and TA 29 are encoded near to a cluster of genes predicted to be involved in the biosynthesis and export of exopolysaccharides which could be linked to biofilm formation in this bacterium (Figure S1B). To determine whether TA systems from ICE*Afe*1 contribute to biofilm formation is to be further analyzed. However, we can not rule out that each system might have a different function under different physiological conditions.

Elucidation of the sequence specificity and thus the cellular targets of each toxin from ICE*Afe*1 might be crucial to determine their role in the physiology of *A. ferrooxidans*.

### Type II TA systems, chromosomal or mobile TA systems?

Hitherto TA systems are classified as chromosomal (stable) or plasmid encoded (mobile). All TA in ICE*Afe*1 are encoded near to transposon-related sequences (Figure S1). Similar distribution occurs with TA from the genomic island of *A. ferrooxidans* ATCC 53993. As it is known that ICEs are modular elements [61], it is possible that TA are part of modules that have been acquired by HGT and contributed to the creation of ICE*Afe*1. Thus we propose that most *A. ferrooxidans* TA systems may belong to either active or inactive MGEs that are inserted in the bacterial chromosome. A similar case has been reported in *Acidithiobacillus caldus*, another acidophilic bacterium [62].

## Supporting Information

Figure S1 **Genetic overview of ICE** ***Afe*** **1 TA and the flanking DNA regions.** The genetic contexts of TA 26 (A), TA 27, TA 28 and TA 29 (B) are indicated. Each gene is colored by COG according to the information on the Integrated Microbial Genomes platform (IMG, http://img.jgi.doe.gov/cgi-bin/w/main.cgi [37]). Color codes of function category for COGs are indicated in the insert below the images.
(TIF)

Figure S2 **Alignment of MazEF-1 system from ICE** ***Afe*** **1 with its ortholog from** ***E. coli*** **.** Protein sequences from toxin (A) and antitoxin (B) were aligned using ClustalW. GenBank accession numbers: MazF_Ec, BAA03918.1; MazF ICEAfe1, YP_002425571.1; MazE_Ec, BAA41177.1; MazE ICEAfe1, YP_002425570.1. Identical and similar amino acids are shown in black and grey, respectively. Functionally important conserved regions [51] are indicated below the MazF and MazE sequences by black lines.
(TIF)

Figure S3 **Alignment of VapC toxins from** ***A. ferrooxidans*** **ATCC 23270.** Protein sequences were aligned using ClustalW. Identical and similar amino acids are shown in black and grey, respectively. The three conserved acidic residues of the PIN-domain are highlighter in green. GenBank accession numbers: VapC-1, YP_002424909; VapC-2, YP_002424974; VapC-3, YP_002425797; VapC-4, YP_002426198; and VapC-5, YP_002426529.
(TIF)

Figure S4 **Effect of ICE** ***Afe*** **1 toxins expression on** ***E. coli*** **CFU.** Cellular growth of *E. coli* BL21(DE3)pLysS cells harboring plasmids containing toxin (T, blue curves) of TA 26 (A), TA 27 (B) and TA 28 (C) post IPTG addition was monitored by measuring the CFU/ml. Cells containing the empty vector (gray curves) were used as a control. The means and standard deviation of two different experiments are plotted.
(TIF)

Figure S5 **ICE** ***Afe*** **1 toxins purification.** Tricine-SDS-PAGE of (His)_6_-tagged toxin proteins purified as it is described at Materials and Methods. The proteins were visualized by staining with Coomassie brilliant blue. The molecular weights of some reference bands (M, PageRuler Unstained Broad Range Protein Ladder, Thermo Scientific) are indicated at the left of the figure.
(TIF)

Figure S6 ***In vitro*** **RNase assay of MazEF-1 system.** 1.6 µg of MS2 RNA was incubated with (+) or without (–) 50 picomoles of the purified MazF-1 toxin and/or MazE-1 antitoxin in 10 mM Tris-HCl (pH 7.8). The reactions were incubated at 37°C for 15 minutes. Lanes 5-7: the reactions contain 100, 150 and 200 picomoles of MazF-1, respectively. Lane 8-10: the reactions contain 100, 150 and 200 picomoles of MazF-1, respectively.
(TIF)

Table S1 **Evolutionary distances among toxins from** ***A. ferrooxidans*** **.**
(XLS)

Supporting Information S1 **Identification of new TA II not describe in TADB.**
(DOCX)

Supporting Information S2 **Structural homologous of PIN domain toxins from** ***A. ferrooxidans*** **.**
(DOCX)

Supporting Information S3 **Gene ID or locus tag of nucleotide sequences used in the phylogenetic analysis.**
(DOCX)
