# Supplementary figures and images for: Toxin-Antitoxin Systems in the Mobile Genome of Acidithiobacillus ferrooxidans
Source: PLoS One. 2014 Nov 10;9(11):e112226. doi: 10.1371/journal.pone.0112226 (PMC4226512; doi:10.1371/journal.pone.0112226)

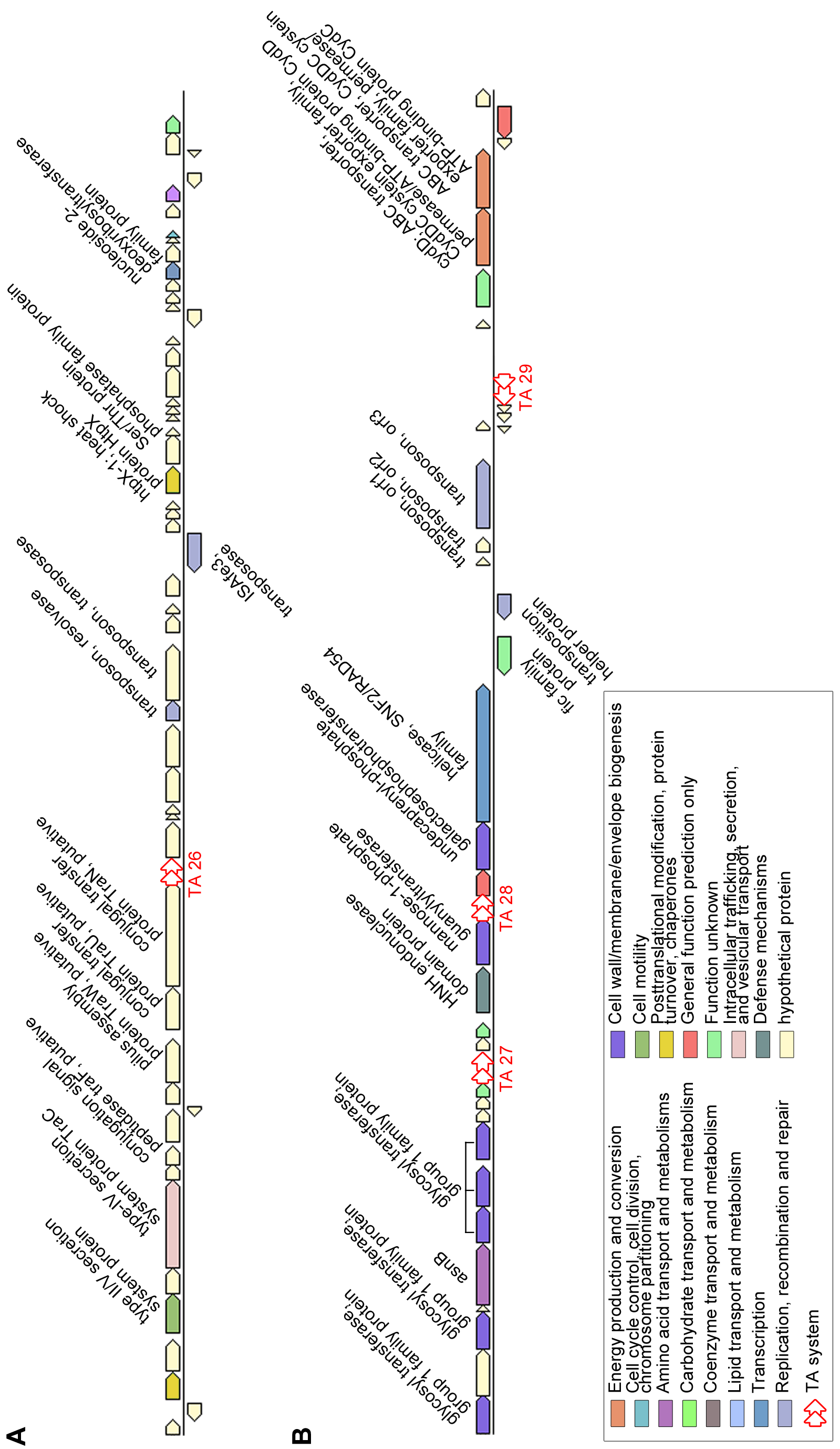

Supplement: Figure S1 — Genetic overview of ICE Afe 1 TA and the flanking DNA regions. The genetic contexts of TA 26 (A), TA 27, TA 28 and TA 29 (B) are indicated. Each gene is colored by COG according to the information on the Integrated Microbial Genomes platform (IMG, http://img.jgi.doe.gov/cgi-bin/w/main.cgi [37]). Color codes of function category for COGs are indicated in the insert below the images. (TIF) [file pone.0112226.s001.tif]

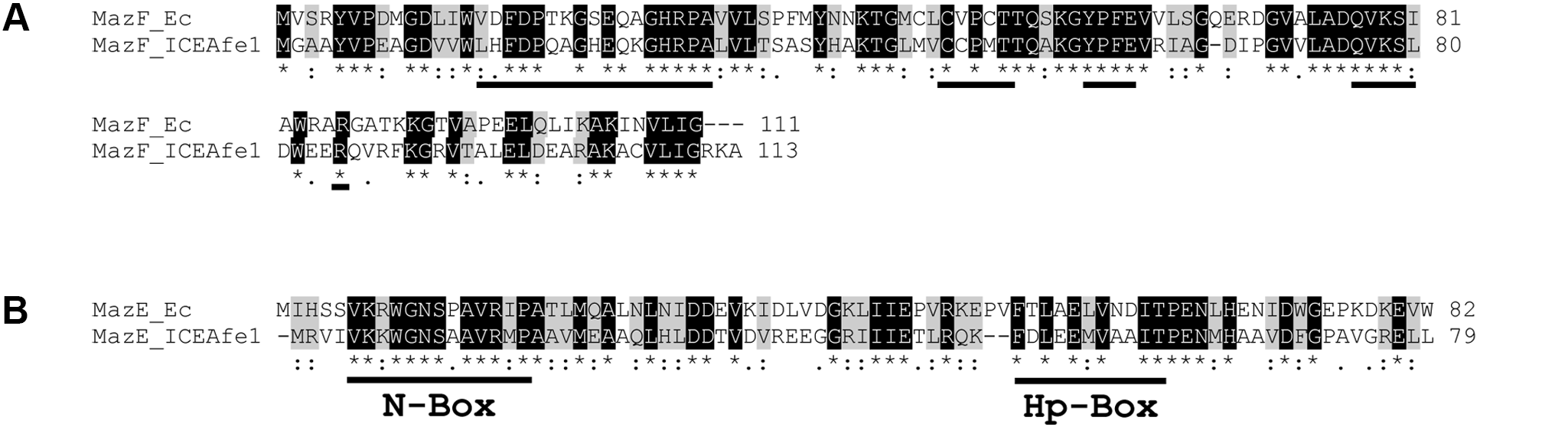

Supplement: Figure S2 — Alignment of MazEF-1 system from ICE Afe 1 with its ortholog from E. coli . Protein sequences from toxin (A) and antitoxin (B) were aligned using ClustalW. GenBank accession numbers: MazF_Ec, BAA03918.1; MazF ICEAfe1, YP_002425571.1; MazE_Ec, BAA41177.1; MazE ICEAfe1, YP_002425570.1. Identical and similar amino acids are shown in black and grey, respectively. Functionally important conserved regions [51] are indicated below the MazF and MazE sequences by black lines. (TIF) [file pone.0112226.s002.tif]

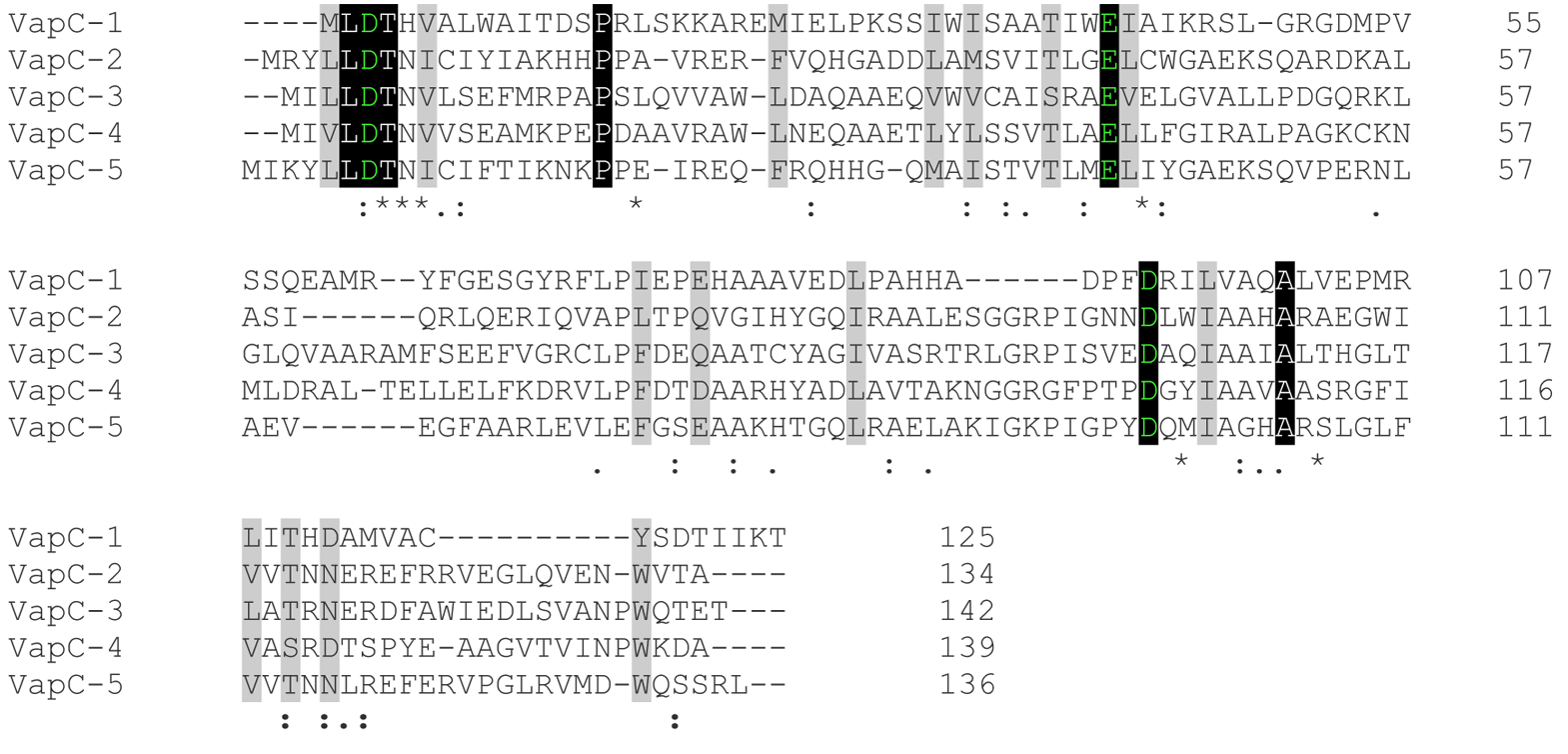

Supplement: Figure S3 — Alignment of VapC toxins from A. ferrooxidans ATCC 23270. Protein sequences were aligned using ClustalW. Identical and similar amino acids are shown in black and grey, respectively. The three conserved acidic residues of the PIN-domain are highlighter in green. GenBank accession numbers: VapC-1, YP_002424909; VapC-2, YP_002424974; VapC-3, YP_002425797; VapC-4, YP_002426198; and VapC-5, YP_002426529. (TIF) [file pone.0112226.s003.tif]

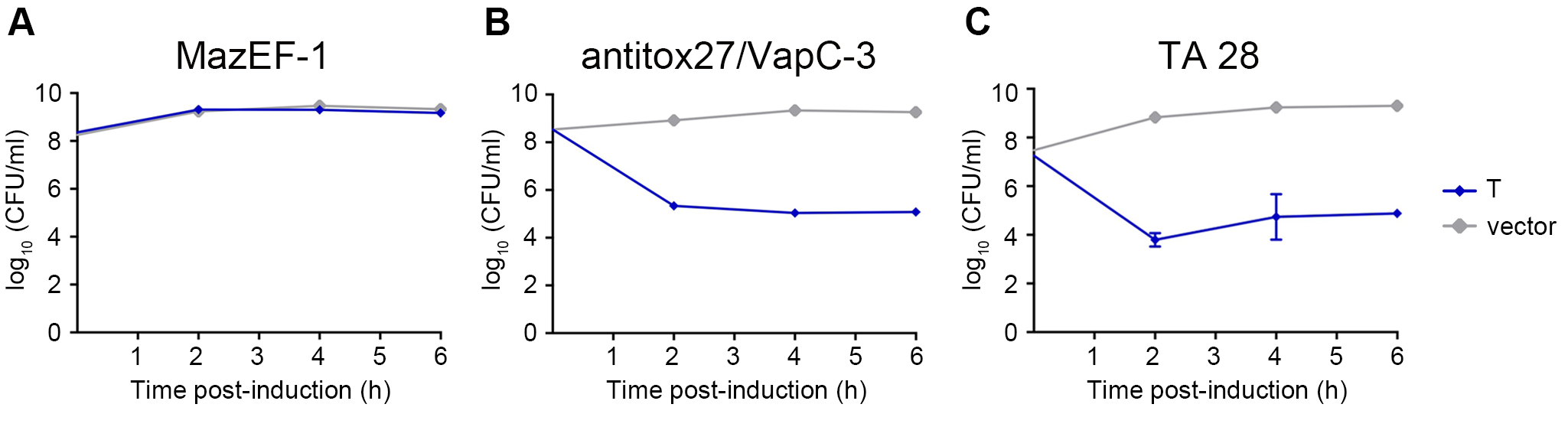

Supplement: Figure S4 — Effect of ICE Afe 1 toxins expression on E. coli CFU. Cellular growth of E. coli BL21(DE3)pLysS cells harboring plasmids containing toxin (T, blue curves) of TA 26 (A), TA 27 (B) and TA 28 (C) post IPTG addition was monitored by measuring the CFU/ml. Cells containing the empty vector (gray curves) were used as a control. The means and standard deviation of two different experiments are plotted. (TIF) [file pone.0112226.s004.tif]

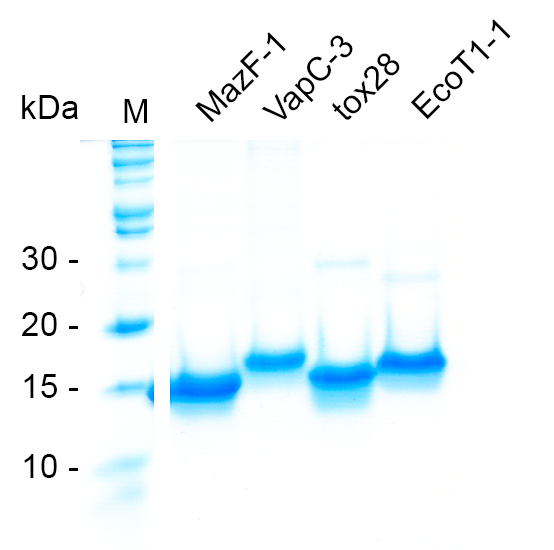

Supplement: Figure S5 — ICE Afe 1 toxins purification. Tricine-SDS-PAGE of (His)6-tagged toxin proteins purified as it is described at Materials and Methods. The proteins were visualized by staining with Coomassie brilliant blue. The molecular weights of some reference bands (M, PageRuler Unstained Broad Range Protein Ladder, Thermo Scientific) are indicated at the left of the figure. (TIF) [file pone.0112226.s005.tif]

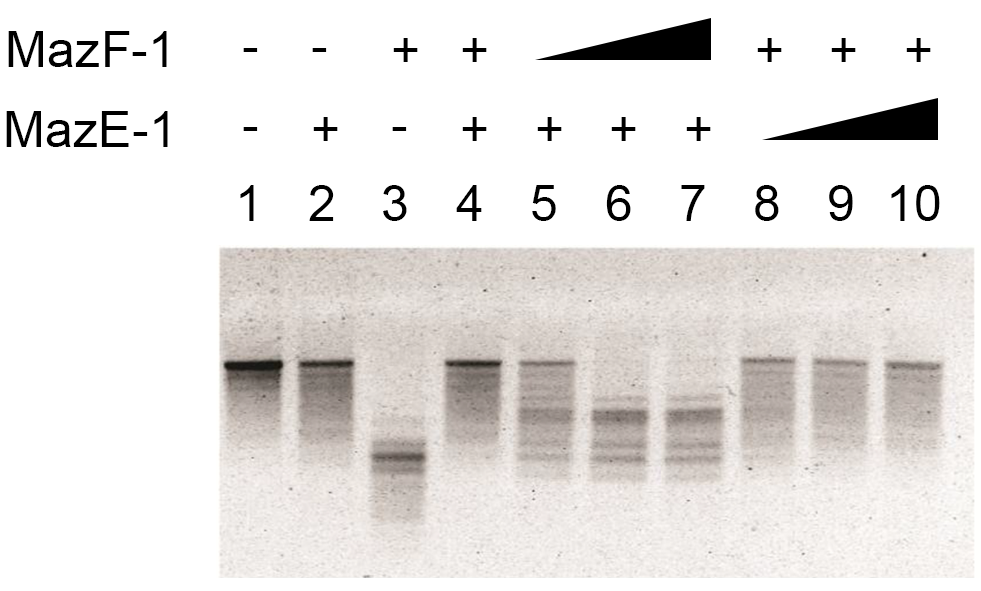

Supplement: Figure S6 — In vitro RNase assay of MazEF-1 system. 1.6 µg of MS2 RNA was incubated with (+) or without (–) 50 picomoles of the purified MazF-1 toxin and/or MazE-1 antitoxin in 10 mM Tris-HCl (pH 7.8). The reactions were incubated at 37°C for 15 minutes. Lanes 5-7: the reactions contain 100, 150 and 200 picomoles of MazF-1, respectively. Lane 8-10: the reactions contain 100, 150 and 200 picomoles of MazF-1, respectively. (TIF) [file pone.0112226.s006.tif]
