# Supplementary material for: Toxin-Antitoxin Systems in the Mobile Genome of Acidithiobacillus ferrooxidans
Source: PLoS One. 2014 Nov 10;9(11):e112226. doi: 10.1371/journal.pone.0112226 (PMC4226512; doi:10.1371/journal.pone.0112226)
Supplement: Supporting Information S1 — Identification of new TA II not describe in TADB. (DOCX) [file pone.0112226.s008.docx]

**Supporting Information S1.** Identification of new TA II not describe in TADB.

TA II 8, 9 and 11. According to TADB Lferr_1137/Lferr_1136, Lferr_0132/Lferr_0133 and Lferr_0234/Lferr_0233 genes encode putative TA systems (TA II 8, 9 and 10, respectively). BLASTP results revealed that the counterpart these systems in the ATCC 23270 strain are encoded by AFE_1418/AFE_1417, AFE_1559/AFE_1560 and AFE_1614/AFE_1613, respectively. So, all these TA II in ATCC 23270 were missing by TADB.

TA II 12. TADB identified as putative TA II one encoded by AFE_1631/AFE_1633 and Lferr_1332/Lferr_1333 genes in ATCC 23270 and ATCC 53993, respectively. According to BLASTP results between both *A. ferrooxidans* strains the protein encoded by AFE_1631 has 99 % identity with the protein encoded by Lferr_1332, so they are counterparts. Conversely, when the same analysis was made with the proteins encoded by AFE_1633 or Lferr_1333, nothing appears with suitable E-values. According to RASTA-Bacteria, Lferr_1331 encodes a putative partner of the protein encoded by Lferr_1332. Both genes are adjacent with 3 intergenic bp in a TA system-like genetic organization. The protein encoded by Lferr_1331 has high identity with addiction module proteins from different bacterial species, but not any on *A. ferrooxidans* ATCC 23270. Also, this putative protein has a conserved domain present in TA systems (upstrm_HI1419 from the superfamily Gp49). So, the correct TA locus in ATCC 53993 seems to be Lferr_1331/Lferr_1332, and was wrongly annotated by TADB. Therefore, we made a TBLASTN search in ATCC 23270 using the protein sequence encoded by Lferr_1331 as search query for its counterpart in this strain. We found a nucleotide region in the ATCC 23270 genome with the potential to encode a protein that is 98 % identical with the query. This region coincides with a open reading frame (Rorf_25786, from bp 1,404,861 to 1,405,109 according to NCBI annotation) identified by RASTA-Bacteria that correspond to the partner of AFE_1631. This region is contained within a gene annotated as pseudo (AFE_1630) because it contains a premature stop codon. Generally, TA systems genes escape genome annotations, in part because small size. So, it is possible that AFE_1630 gene has been poorly annotated and in reality corresponds to a gene encoding a TA protein having as a partner the one encoded by AFE_1631.

TA II 13. According to TADB, Lferr_0263/Lferr_0264 genes encode a putative TA system in ATCC 53993. BLASTP results revealed that the protein encode by Lferr_0263 is 78 % identical with a protein encode by AFE_1700, but with Lferr_0264 nothing appears in *A. ferrooxidans* ATCC 23270 with suitable E-values. TBLASTN results with Lferr_0264 as query reveals that there are 62 % identity with a region from bp 1,474,883 to 1,475,077 in ATCC 23270. This corresponds to a pseudogene (AFE_1701).

TA II 14. According to RASTA-Bacteria a putative TA II is encoded by Lferr_1422/Lferr_1423, but this system do not appear in the TADB data. BLASTP results revealed that the protein encoded by Lferr_1422 is 100 % identical with a protein encoded by AFE_1732 gene, and that encoded by Lferr_1423 is 100 % identical with a protein encoded by AFE_1733 gene. This TA II was missed by TADB in ATCC 53993 strain, but not in the other strain.

TA II 17. Lferr_2046/Lferr_2045 encode a putative TA II identified by TADB in ATCC 53993. BLASTP results revealed that the proteins encoded by these genes are 100 % identical with the proteins encoded by AFE_2415 and AFE_2414 genes in ATCC 23270, respectively. Thus, this TA II was missed by TADB in this strain.

TA II 18. According to TADB, Lferr_2283/Lferr_2284 genes encode a putative TA system in ATCC 53993. BLASTP results revealed that the protein encoded by Lferr_2284 is 100 % identical to a protein encoded by AFE_2658 gene. However, we did not find a counterpart to the protein encoded by Lferr_2283 with a suitable E-value on the other strain. When we looked at the genetic region where Lferr_2283/Lferr_2284 are encoded and compared it with the other strain, it was obvious that the counterpart gene of Lferr_2283 was missing in ATCC 23270. A BLASTN search on ATCC 23270 with the nucleotide sequence encoding Lferr_2283 and Lferr_2284 as query (bp 2,255,703 to 2,256,177, according to NCBI annotation) reveals 100 % identity between them. So, the gene that is the TA partner of AFE_2658 in ATCC 23270 was not annotated. We named this gene AFE_2657’ and it is encoded from bp 2,361,002 to 2,361,202.

TA II 19. TADB identified a TA II one encoded by Lferr_2392/Lferr_2391 in ATCC 53993. BLASTP results reveals that the protein encoded by Lferr_2392 is 100 % identical to the protein encoded by AFE_2771. On the other hand, the protein encoded by Lferr_2391 is 80 % identical with the protein encoded by AFE_2655. Both genes from ATCC 23270 (AFE_2771 and AFE_2655) cannot be a TA II because these genes are separated by 5 kbp. TBLASTN results using the coding region of Lferr_2391/Lferr_2392 as query (from bp 2,39,455 to 2,360,099) reveals that in ATCC 23270 there is a region 100 % identical. Inside this region there is an annotated pseudogene (AFE_2770).

TA II 22. According to TADB AFE_2981/AFE_2982 genes in *A. ferrooxidans* ATCC 23270 encoded a putative TA II. BLASTP results indicated that the protein encoded by AFE_2981 has 88 % identity with the protein encoded by Lferr_2590 on the other strain, but when the same analysis was conducted with the protein encoded by AFE_2982, nothing appears with an E-value bellow 1.3. When we looked at the genetic region where AFE_2981/AFE_2982 are encoded and compared the genetic context on the other strain, it was evident that two genes are missing in ATCC 53993, corresponding to the counterparts of AFE_2981 and AFE_2982. A BLASTN search in ATCC 53993 with the nucleotide region encoding AFE_2981 and AFE_2982 (from bp 2,671,270 to bp 2,671,529) as query reveals that there is 100 % identity between them. So, the counterparts of AFE_2981 and AFE_2982 in ATCC 53993 were not annotated and they probably correspond to a missing TA II. We named these genes Lferr_2595’ and Lferr_2595’’ and are encoded from bp 2,571,231 to bp 2,571,368.

TA II 28. This system was identified by Rasta-Bacteria and does not appear in TADB. It is exclusive of ATCC 23270 strain.

TA II 29. TADB reports the gene AFE_1383 as part of two possible TA systems (the combinations AFE_1383/AFE_1382 and AFE_1384/AFE_1383). In these cases the gene AFE_1383 is shared. The protein encoded by this gene has a HTH_XRE conserved domain, usually present in antitoxins. To decipher which is the partner of AFE_1383 we cloned both combinations of genes to carried out a functional analysis of TA II. The overexpression of AFE_1382 does not affect the *E. coli* growth. Conversely, AFE_1384 could not be cloned in the absent of AFE_1383 because its toxicity. When AFE_1384 was cloned in the present of AFE_1383, the bacteria growth normally and both proteins are overexpressed. Thus, we determined that the right TA II is encoded by AFE_1383/AFE_1384.
