# Supplementary material for: Toxin-Antitoxin Systems in the Mobile Genome of Acidithiobacillus ferrooxidans
Source: PLoS One. 2014 Nov 10;9(11):e112226. doi: 10.1371/journal.pone.0112226 (PMC4226512; doi:10.1371/journal.pone.0112226)
Supplement: Supporting Information S2 — Structural homologous of PIN domain toxins from A. ferrooxidans . (DOCX) [file pone.0112226.s009.docx]

**Supporting information S2.** Structural homologous of PIN domain toxins from *A. ferrooxidans*.

| **Toxin (locus)** | **Structural homologue^a^** | **Organism** | **PDB ID** | **% id** | **Ref** |
| --- | --- | --- | --- | --- | --- |
| tox1 (AFE_0086) | conserved hypothetical protein pae0151 | *Pyrobaculum aerophilum* | 2FE1 | 20 | [1] |
| tox10 (AFE_1578) | rv0301 rv0300 toxin antitoxin complex from mycobacterium tuberculosis | *Mycobacterium tuberculosis* | 3H87 | 19 | [2] |
| tox11 (AFE_1613) | rna(fmet)-specific endonuclease vapc | *Shigella flexneri* | 3TND | 12 | [3] |
| tox19 (Lferr_2391) | rna(fmet)-specific endonuclease vapc | *Shigella flexneri* | 3TND | 38 | [3] |
| tox23 (AFE_2984) | conserved hypothetical protein pae0151 | *Pyrobaculum aerophilum* | 2FE1 | 21 | [1] |
| tox24 (AFE_3173) | conserved hypothetical protein pae0151 | *Pyrobaculum aerophilum* | 2FE1 | 18 | [1] |
| tox25 (AFE_3269) | conserved hypothetical protein pae0151 | *Pyrobaculum aerophilum* | 2FE1 | 19 | [1] |

All the structural model were generated with > 99 % confidence and >85 % coverage with the corresponding templates using Phyre 2.0 server [4].

^a^PDB molecule.

**References**

1. Bunker RD, McKenzie JL, Baker EN, Arcus VL (2008) Crystal structure of PAE0151 from Pyrobaculum aerophilum, a PIN-domain (VapC) protein from a toxin-antitoxin operon. Proteins 72: 510–518. doi:10.1002/prot.22048.

2. Min AB, Miallau L, Sawaya MR, Habel J, Cascio D, et al. (2012) The crystal structure of the Rv0301-Rv0300 VapBC-3 toxin-antitoxin complex from M. tuberculosis reveals a Mg^2+^ ion in the active site and a putative RNA-binding site. Protein Sci Publ Protein Soc 21: 1754–1767. doi:10.1002/pro.2161.

3. Dienemann C, Bøggild A, Winther KS, Gerdes K, Brodersen DE (2011) Crystal structure of the VapBC toxin-antitoxin complex from Shigella flexneri reveals a hetero-octameric DNA-binding assembly. J Mol Biol 414: 713–722. doi:10.1016/j.jmb.2011.10.024.

4. Kelley LA, Sternberg MJE (2009) Protein structure prediction on the Web: a case study using the Phyre server. Nat Protoc 4: 363–371. doi:10.1038/nprot.2009.2.
