# Supplementary material for: Toxin-Antitoxin Systems in the Mobile Genome of Acidithiobacillus ferrooxidans
Source: PLoS One. 2014 Nov 10;9(11):e112226. doi: 10.1371/journal.pone.0112226 (PMC4226512; doi:10.1371/journal.pone.0112226)
Supplement: Supporting Information S3 — Gene ID or locus tag of nucleotide sequences used in the phylogenetic analysis. (DOCX) [file pone.0112226.s010.docx]

**Supporting information S3:** Gene ID or locus tag of nucleotide sequences used in the phylogenetic analysis.

|  | Gene ID or locus tag* |
| --- | --- |
| AFE_0086_tox1_Acidithiobacillus ferrooxidans ATCC 23270 | 7136859 |
| AFE_0413_VapC-1_tox3_Acidithiobacillus ferrooxidans ATCC 23270 | 7134445 |
| AFE_0478_VapC-2_tox4_Acidithiobacillus ferrooxidans ATCC 23270 | 7135410 |
| AFE_0870_YoeB-1_tox5_Acidithiobacillus ferrooxidans ATCC 23270 | 7135240 |
| AFE_0890_StbE-1_tox6_Acidithiobacillus ferrooxidans ATCC 23270 | 7134722 |
| AFE_1099_MazF-1_tox26_Acidithiobacillus ferrooxidans ATCC 23270 | 7134427 |
| AFE_1362_VapC-3_tox27_Acidithiobacillus ferrooxidans ATCC 23270 | 7135145 |
| AFE_1368_tox28_Acidithiobacillus ferrooxidans ATCC 23270 | 7134927 |
| AFE_1384_EcoT1-1_tox29_Acidithiobacillus ferrooxidans ATCC 23270 | 7135287 |
| AFE_1412_tox7_Acidithiobacillus ferrooxidans ATCC 23270 | 7135869 |
| AFE_1417_tox8_Acidithiobacillus ferrooxidans ATCC 23270 | 7134672 |
| AFE_1559_tox9_Acidithiobacillus ferrooxidans ATCC 23270 | 7134497 |
| AFE_1578_tox10_Acidithiobacillus ferrooxidans ATCC 23270 | 7136872 |
| AFE_1613_tox11_Acidithiobacillus ferrooxidans ATCC 23270 | 7136703 |
| AFE_1733_tox14_Acidithiobacillus ferrooxidans ATCC 23270 | 7134696 |
| AFE_2129_VapC-5_tox16_Acidithiobacillus ferrooxidans ATCC 23270 | 7135833 |
| AFE_2414_tox17_Acidithiobacillus ferrooxidans ATCC 23270 | 7134212 |
| AFE_2888_NspT2-1_tox21_Acidithiobacillus ferrooxidans ATCC 23270 | 7134524 |
| AFE_2984_tox23_Acidithiobacillus ferrooxidans ATCC 23270 | 7136298 |
| AFE_3173_tox24_Acidithiobacillus ferrooxidans ATCC 23270 | 7135977 |
| AFE_3269_tox25_Acidithiobacillus ferrooxidans ATCC 23270 | 7134684 |
| Rorf_25786_tox12_Acidithiobacillus ferrooxidans ATCC 23270 | ^(a)^ |
| tox19_Acidithiobacillus ferrooxidans ATCC 23270 | ^(b)^ |
| CcdB-F_Escherichia coli K-12 plasmid F DNA | 1263593 |
| CcdB-O157_Escherichia coli O157:H7 str. EDL933 | 956724 |
| ChpBK_Escherichia coli E24377A | 5585980 |
| hypothetical protein_Firmicutes bacterium ASF500 | N510_03025 |
| hypothetical protein_Gallionella sp SCGC AAA018-N21 | A37I_02600 |
| hypothetical protein_Klebsiella pneumoniae BIDMC 24 | L460_05131 |
| MazF_Escherichia coli 536 | 4191662 |
| MazF_Haemophilus parasuis MN-H KuehnKR3 | HPSMNH_1803 |
| PemK-1_Acidithiobacillus ferrooxidans ATCC 23270 | 7136919 |
| PemK-1_Acidithiobacillus ferrooxidans ATCC 53993 | 6878503 |
| PemK_Kid_Escherichia coli plasmid NR1 | 4924840 |
| PemK-like protein_Sphingobium japonicum UT26S | 8955169 |
| toxin_Lamprocystis purpurea DSM 4197 | A39O_16275 |
| MazE_toxin_Acidithiobacillus ferrivorans SS3 | 11043687 |
| YdcE_Bacillus subtilis subsp. subtilis str. 168 | 939935 |
| AFE_0088_HigB-1_Acidithiobacillus ferrooxidans ATCC 23270 | 7136176 |
| AFE_2982_CcrT1-1_Acidithiobacillus ferrooxidans ATCC 23270 | 7134296 |
| EcoT1-EDL933_Escherichia coli O157:H7 str. EDL933 | 960786 |
| Acife_2254_Acidithiobacillus ferrivorans SS3 | 11044452 |
| Galf_2192_Gallionella capsiferriformans ES-2 | 9613556 |
| Oscil6304_5031_Oscillatoria acuminata PCC 6304 | 14110405 |
| ROD_36431_Citrobacter rodentium ICC168 | 8713198 |
| Lferr_0090_HigB-1_Acidithiobacillus ferrooxidans ATCC 53993 | 6876040 |
| Lferr_0995_YoeB-1_Acidithiobacillus ferrooxidans ATCC 53993 | 6876962 |
| Lferr_1012_StbE-1_Acidithiobacillus ferrooxidans ATCC 53993 | 6876979 |
| ParE1_Escherichia coli O157:H7 str. Sakai | 914057 |
| ParE3_Escherichia coli O157:H7 str. Sakai chromosome | 914563 |
| ParE-RK2_Plasmid RK2_Klebsiella pneumoniae | ^(c)^ |
| PasB_Acidithiobacillus caldus plasmid pTC-F14 | 1076413 |
| RelE-307_Escherichia coli B171 plasmid pB171 | 1238646 |
| RelE-K12_Escherichia coli str. K-12 substr. MG1655 | 947549 |
| StbE-Morg_Morganella morganii strain M203 plasmid R485 | 11179237 |
| StbE-Salm_Salmonella enterica subsp. enterica serovar Enteritidis pSE34 | pSE34_02 |
| toxin RelE_Acidithiobacillus thiooxidans ATCC 19377 | AthiA1_010100002662 |
| Txe_Enterococcus faecium U37 plasmid pRUM | 4594985 |
| YafQ_Escherichia coli str. K-12 substr. MG1655 | 944911 |
| YoeB_Escherichia coli str. K-12 substr. MG1655 | 1450274 |
| AFE_1780_VapC-4_tox19_Acidithiobacillus ferrooxidans ATCC 23270 | 7134280 |
| hypothetical protein_Lamprocystis purpurea DSM 4197 A39ODRAFT | A39O_14060 |
| hypothetical protein_Pseudomonas aeruginosa GM41 | PMI27_01882 |
| Lferr_0576_VapC-1_Acidithiobacillus ferrooxidans ATCC 53993 | 6876538 |
| Lferr_0638_VapC-2_Acidithiobacillus ferrooxidans ATCC 53993 | 6876600 |
| Lferr_1456_VapC-4_Acidithiobacillus ferrooxidans ATCC 53993 | 6877430 |
| Lferr_1788_VapC-5_Acidithiobacillus ferrooxidans ATCC 53993 | 6877771 |
| MvpT_Shigella flexneri plasmid pMYSH6000 | ^(d)^ |
| PilT_Acidithiobacillus ferrivorans SS3 | 11043197 |
| PilT_Desulfotignum phosphitoxidans DSM 13687 | Dpo_8c01680 |
| PilT_Burkholderia xenovorans LB400 | 4007622 |
| VapC_Leptospira interrogans serovar Copenhageni str. Fiocruz L1-130 | 2769615 |

* Gene ID and locus tag are according to NCBI; ^(a)^ GenBank CP001219.1: bp 1,404,861 to 1,405,109; ^(b)^ GenBank CP001219.1: bp 2,361,002 to 2,361,205; ^(c)^ GenBank L05507: bp 431 to 742; ^(d)^ GenBank U82621: complement bp 3,605 to 3,207.
